# NRF2 in hypertrophic scarring: A stage-dependent redox hub poised between resolution and persistence

**DOI:** 10.1016/j.redox.2026.104460

**Published:** 2026-10-27

**Authors:** Weerawon Thangboonjit, Mutita Pluempreecha, Tanyapohn Soingam, Uraiwan Panich

**Affiliations:** Department of Pharmacology, Faculty of Medicine Siriraj Hospital, Mahidol University, Bangkok, 10700, Thailand

**Keywords:** Hypertrophic scarring, KEAP1–NRF2 signaling, Redox signaling, Myofibroblast persistence, Ferroptosis, Mechanoredox remodeling

## Abstract

Hypertrophic scarring (HTS) is a fibroproliferative outcome of deep dermal injury, reflecting failed scar resolution rather than collagen excess alone. Sustained oxidative and inflammatory signaling is a prominent feature of HTS. Reactive oxygen species (ROS) amplify TGF-β/Smad and NF-κB-linked signaling, reinforce matrix stiffening, and sustain activated fibroblasts within a mechanically loaded niche. However, how NRF2 activity changes across scar maturation—and whether it remains protective, represents a compensatory response, or supports fibroblast persistence—remains unresolved. The KEAP1–NRF2 pathway is a major redox-regulatory node, coordinating antioxidant, detoxification, metabolic, proteostatic, and anti-ferroptotic programs. We propose a testable, stage- and compartment-dependent model rather than a fixed early-to-late switch. Early injury may preferentially engage the redox-sensitive Neh2–KEAP1 arm, whereas growth-factor and mechanical signaling in established scars may increasingly influence NRF2 through the KEAP1-independent Neh6–GSK-3β/β-TrCP arm. Autophagy-linked phospho-p62 (SQSTM1) may provide a separate noncanonical but KEAP1-dependent route to NRF2 stabilization. We synthesize TGF-β/Smad–NOX4 signaling, NF-κB inflammation, p62 feedback, iron–lipid redox and ferroptosis, and mechanoredox coupling—and apply a non-ordinal engagement-to-phenotype (E2P) framework considering disease proximity, NRF2 engagement, causal dependence, scar-relevant phenotype, and replication/methodological quality. Because stage- and cell-resolved nuclear NRF2 data in human HTS are lacking, and disease-proximal evidence for persistence-permissive activity remains limited despite a recent independent gain-/loss-of-function study in human HTS fibroblasts, NRF2 is best viewed as a conditional redox hub rather than a uniformly antifibrotic switch. This framework identifies experiments needed to resolve NRF2 directionality and determine whether stage- and compartment-aware local modulation is therapeutically justified.

## Highlights

• NRF2 is a conditional redox-regulatory node, not a uniformly antifibrotic switch.
• Dual Neh2–KEAP1 and Neh6–GSK-3β/β-TrCP control: a testable stage-dependent model.
• E2P criteria distinguish NRF2 engagement from nonspecific antioxidant effects.
• Persistence-permissive NRF2 in established HTS is hypothetical, on limited evidence.
• Matrix stiffness may stabilize NRF2 via Neh6, uncoupling it from oxidative stress.

## Graphical abstract

**NRF2 as a stage-dependent redox hub in hypertrophic scarring.** Overview of the review's central thesis. In early injury, an oxidative and electrophilic burst may preferentially engage the redox-sensitive Neh2–KEAP1 arm, permitting transient NRF2 activation that may limit inflammation and fibroblast priming and favor resolution. As the scar matures and becomes established, growth-factor and mechanical inputs may increase the contribution of the KEAP1-independent Neh6–GSK-3β/β-TrCP arm, while autophagy-linked p62–KEAP1 signaling provides a separate noncanonical but KEAP1-dependent route to NRF2 stabilization. The NRF2 state in established scars may reflect insufficient pathway activity, compensatory/pro-resolution activation, or persistence-permissive activity. Any therapeutic implication should remain conditional on human pathway directionality, target cell type, scar phenotype, and local pathway engagement rather than a fixed early-activation/late-restraint strategy. The model is presented as a testable hypothesis; the supporting disease-proximal evidence remains limited (Sections 3.2 and 4.4).

**Figure:**
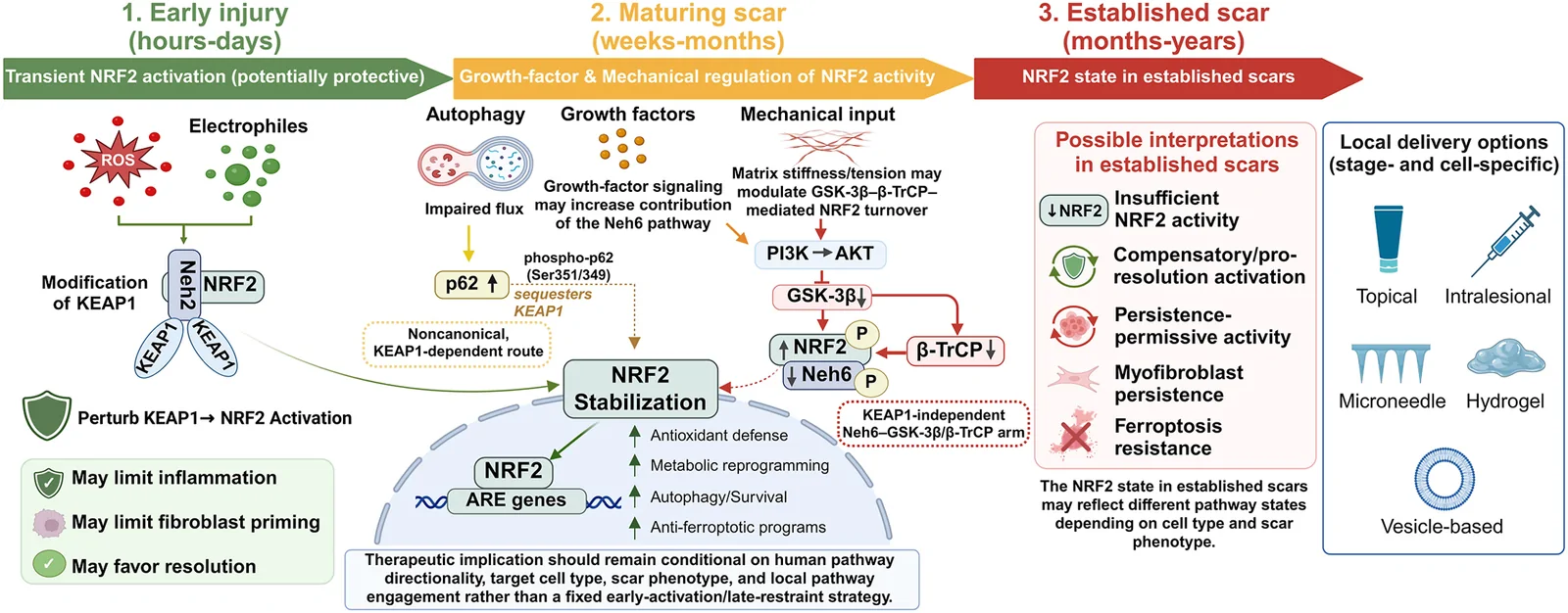

## 1 Introduction

Hypertrophic scarring (HTS) is a common fibroproliferative outcome of deep dermal injury from burns, surgery, trauma, and inflammatory dermatoses. Clinically, it presents as a raised, erythematous scar confined to the original wound boundaries, distinguishing it from keloids, which extend beyond the injury margins [1], [2]. Unlike mature scars that progressively flatten and soften, HTS may persist with pruritus, pain, stiffness, contracture, and functional and psychosocial morbidity, with burn-related HTS among the most pervasive late complications of burn injury [2], [3], [4], [5]. For brevity, HTS denotes both the process and the lesion, resolved by context; keloid evidence is invoked only where it offers mechanistic insight into redox- or NRF2-linked pathways plausibly relevant to HTS, and is interpreted cautiously because keloids and HTS differ in margin behavior, recurrence, mechanobiology, and hormonal responsiveness [1], [4]. Throughout this review, findings derived from keloids or other non-HTS fibrotic systems are explicitly identified and are used to support mechanistic plausibility rather than as direct evidence of HTS-specific NRF2 regulation. Current scar care—tension reduction, hydration, photoprotection, silicone and pressure therapy, intralesional corticosteroids with or without 5-fluorouracil, laser, and surgical revision—improves outcomes but incompletely addresses the upstream mechanisms that drive fibroinflammation, myofibroblast persistence, and aberrant matrix remodeling [1], [6], [7], [8], [9], [10].

At a mechanistic level, HTS is better viewed as a failure of scar resolution than as collagen excess alone. Sustained inflammatory signaling, persistent fibroblast and myofibroblast activation, excessive extracellular matrix (ECM) deposition, and dysregulated remodeling form a self-reinforcing fibroinflammatory niche [1], [11], [12], and mechanical cues amplify it: wound tension and matrix stiffening reinforce profibrotic transcription and sustain myofibroblast survival and matrix output [11], [13], [14], [15]. Redox dysregulation is a recurrent and arguably proximal feature of this niche. Persistent or excessive reactive oxygen species (ROS) promote skin fibrosis by amplifying TGF-β/Smad signaling, reinforcing NF-κB-linked inflammatory circuits, and shifting the balance between fibroblast resolution and persistence [16], [17], [18], [19], [20]. Human and experimental studies consistently identify oxidative stress in fibrotic skin, motivating redox control as a therapeutic axis [16], [19], [21].

The Kelch-like ECH-associated protein 1–nuclear factor erythroid 2-related factor 2 (KEAP1–NRF2) pathway is a major redox-regulatory node in this setting, coordinating antioxidant defense, electrophile and xenobiotic detoxification, metabolic adaptation, proteostasis, mitochondrial quality control, iron handling, and inflammatory restraint [22], [23], [24], [25]. Under basal conditions, KEAP1 drives NRF2 ubiquitination and degradation; oxidative or electrophilic stress relieves this repression, allowing NRF2 stabilization, nuclear accumulation, and antioxidant response element (ARE)-driven transcription [21], [26], [27]. Crucially, NRF2 biology is context-dependent: activation can protect tissue by limiting oxidative injury and inflammatory amplification, yet sustained or cell-type-restricted NRF2 signaling can instead confer stress tolerance, reshape matrix remodeling, and support cell persistence in chronically injured tissue [24], [28], [29], [30].

This duality is not new to biology in general. A substantial oncology literature describes a “dark side” of NRF2, in which a cytoprotective program that can prevent injury in normal cells may confer chemoresistance and a survival advantage when constitutively active in established tumors [31], [32]. What this review adds for HTS is not the observation that NRF2 can be double-edged—that principle is established—but a disease-specific molecular framework for *how NRF2 regulation may be organized in scar tissue and a route to test it directly*. The oncology literature largely attributes this functional shift to somatic KEAP1/NRF2/CUL3 mutations, a mechanism with no known counterpart in HTS, which is a non-neoplastic, non-mutational disease. We propose instead that, in HTS, a comparable functional shift could arise physiologically through context-dependent changes in the relative contribution of NRF2 degradation routes as the wound matures: the redox-sensitive Neh2–KEAP1 arm may be favored during early oxidative/electrophilic stress, whereas the Neh6–GSK-3β/β-TrCP arm may become more influential under sustained growth-factor and mechanical signaling. This temporal organization has not been directly demonstrated in the same scar-relevant cell population and should therefore be regarded as a testable inference rather than an observed switch. This framework generates two contributions beyond the oncology paradigm: a potential mechanistic link from matrix stiffness itself to NRF2 stabilization (Section 4.5), and a falsifiable, stage-resolved prediction—rather than a retrospective correlation with mutation status—for whether NRF2 activity may shift from predominantly protective toward persistence-permissive behavior.

Prior syntheses have treated HTS pathobiology, oxidative stress in skin fibrosis, NRF2 biology, and scar management largely in isolation [1], [6], [24], [33], [34]. The two most directly relevant—Hiebert and Werner's account of NRF2 in wound healing [28] and Cuadrado et al.’s pharmacological synthesis of the KEAP1–NRF2 partnership in chronic disease [24]—establish, respectively, that NRF2 does more than provide cytoprotection during repair and that the pathway is therapeutically tractable across organs. Together with the broader cutaneous-fibrosis literature discussed below, these studies establish that NRF2 involvement in wound repair and fibrotic redox biology is not itself novel; the contribution of the present review is the HTS-specific organization of this evidence by scar stage, cellular compartment, regulatory route, and evidential strength. Neither, however, resolves NRF2 activity by scar stage, cellular compartment, and degradation route simultaneously; neither applies an explicit attribution standard to separate genuine NRF2 involvement from nonspecific antioxidant effects in the scar literature; and neither derives a mechanoredox route by which matrix stiffness could stabilize NRF2 independently of KEAP1.

This review supplies that synthesis. We organize the redox pathobiology of HTS around the KEAP1–NRF2 axis and its crosstalk with the profibrotic, inflammatory, proteostatic, ferroptotic, and mechanoredox circuits that build and sustain the scar (Fig. 1). Because several mechanistic chains connect steps demonstrated in scar-relevant cells with steps established mainly in other tissues, we explicitly separate demonstrated evidence from inference. Rather than presenting NRF2 as a uniformly protective antioxidant pathway, this review develops a stage- and compartment-resolved model in which NRF2 activity may either support scar resolution or permit scar persistence, depending on the regulatory route engaged and the cellular context.

**Fig. 1:**
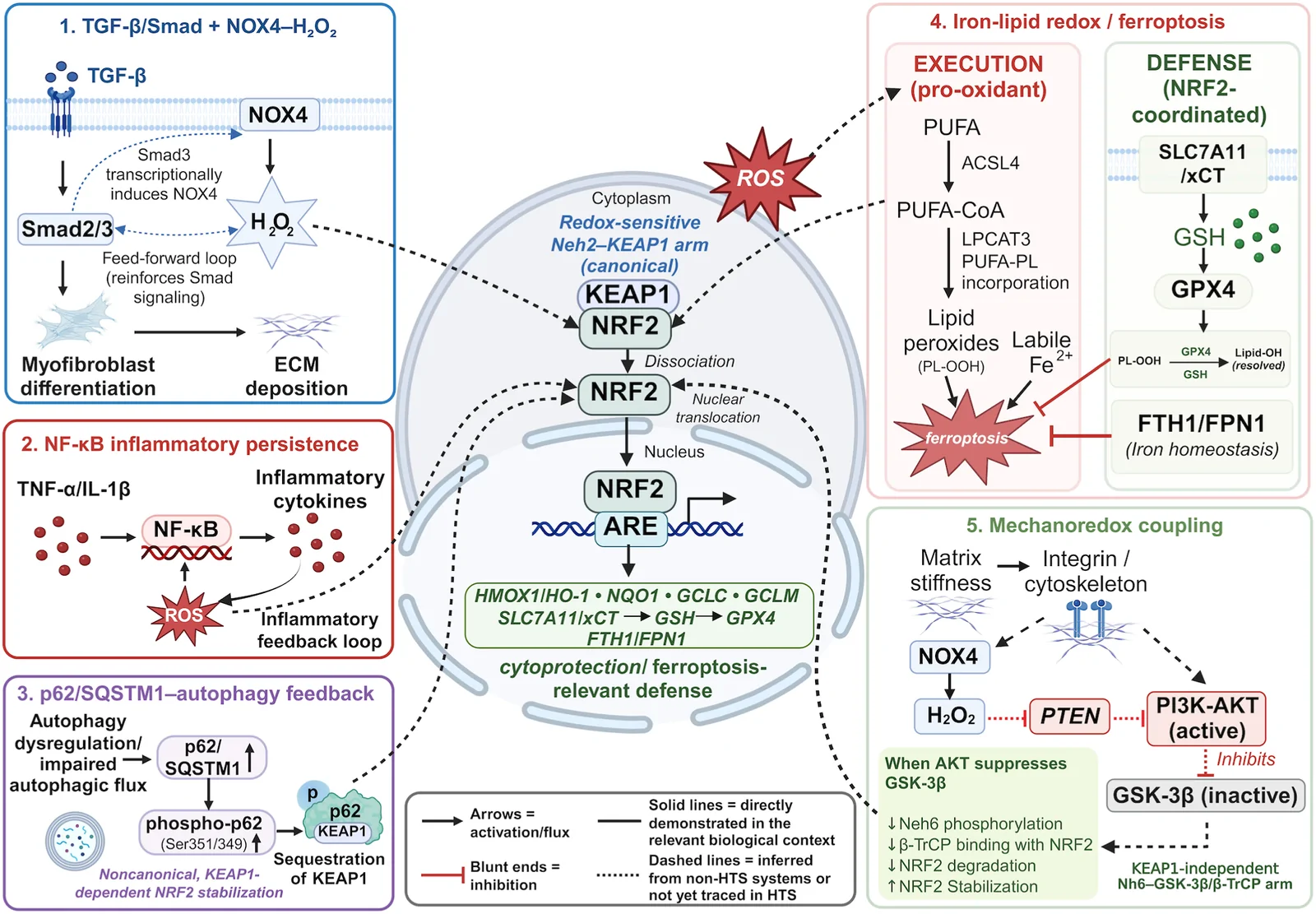
**Redox-mechanistic modules converging on the KEAP1–NRF2–ARE axis in hypertrophic scarring.** The KEAP1–NRF2 axis integrates oxidative and electrophilic inputs to drive NRF2-coordinated cytoprotective and ferroptosis-relevant programs. Five interacting circuits developed in Section 4 are shown: (1) TGF-β/Smad differentiation with the NOX4–H_2_O_2_ feed-forward loop; (2) NF-κB-linked inflammatory persistence; (3) p62–autophagy feedback driving noncanonical but KEAP1-dependent NRF2 stabilization; (4) iron–lipid redox and ferroptosis, separating the execution machinery (ACSL4/LPCAT3 and labile iron) from the NRF2-coordinated defense machinery; and (5) mechanoredox coupling, in which matrix stiffness and tension feed both ROS generation and KEAP1-independent NRF2 stabilization. The Neh2–KEAP1 and Neh6–GSK-3β/β-TrCP arms are therefore distinguished from the separate p62–KEAP1 route. The functional consequence remains context-dependent: redox-driven NRF2 may favor resolution in early repair, whereas whether sustained NRF2 in established scars is protective/compensatory or persistence-permissive remains unresolved. Arrows denote activation or flux, and blunt ends denote inhibition. Solid connections denote relationships directly demonstrated in the relevant biological context; dashed connections denote relationships inferred from non-HTS systems or not yet traced in HTS, including the NOX4/PTEN–PI3K–AKT–GSK-3β–Neh6–NRF2 sequence, which remains a mechanistic prediction in scar fibroblasts. H_2_O_2_ is specified where NOX4/peroxide signaling is intended rather than using ROS generically. Colour is not used as the sole carrier of meaning, and line style and labels remain distinguishable in grayscale and for readers with colour-vision deficiency; abbreviations are defined in the main text.

The conceptual core, developed in Section 2, integrates established NRF2 regulatory mechanisms into a proposed dual-degron logic. The Neh2–KEAP1 and Neh6–GSK-3β/β-TrCP pathways are mechanistically established; however, their relative stage-specific contributions in HTS have not been directly demonstrated. We hypothesize that the oxidative and electrophilic environment of early injury may preferentially engage the Neh2–KEAP1 arm, whereas sustained growth-factor and mechanical signaling in established scars may increasingly influence NRF2 through the KEAP1-independent Neh6–GSK-3β/β-TrCP pathway. In parallel, autophagy-linked phospho-p62–mediated KEAP1 sequestration may provide a distinct noncanonical but KEAP1-dependent route to NRF2 stabilization. Thus, the proposed temporal organization of these regulatory mechanisms in HTS remains an inference requiring direct experimental validation. This framework predicts, rather than establishes, a stage-dependent shift of function. Early or transient NRF2 engagement may restrain oxidative-inflammatory amplification and fibroblast priming; sustained NRF2 activity in established scars may instead enhance stress tolerance, reinforce anti-ferroptotic defenses, and support activated-fibroblast survival—a state we term persistence-permissive NRF2 activity—a designation that refers specifically to increased stress tolerance and survival of already-activated myofibroblasts, and is not meant to imply that NRF2 initiates fibrosis or that all NRF2-supported cell survival during repair is undesirable. We treat this as a working model, not as a settled conclusion. The opposing interpretation—that NRF2 remains net pro-resolution even in established scars—is plausible and requires direct testing in stage-annotated human HTS tissue and controlled scar models. Its central directional claim currently rests on a small and largely single-group experimental literature (Section 4.4), and we indicate throughout where conclusions await confirmation in stage-annotated human HTS tissue. Nevertheless, an independent study using Nrf2 gain- and loss-of-function in human HTS fibroblasts supports a role for NRF2 in fibroblast behavior, although it does not resolve stage-specific nuclear NRF2 directionality in human scar tissue or establish the proposed stiffness–Neh6 mechanism [35].

For terminology, ‘protective’ is used broadly for reduced tissue injury, ‘pro-resolution’ for processes that promote successful termination of inflammation and repair, and ‘antifibrotic’ for direct reduction of fibrogenic cell activity or pathological extracellular-matrix accumulation. Scar stage is likewise treated as a continuous, overlapping biological state rather than a deterministic clock: broad temporal descriptors are used only as guides and should be paired with objective phenotype, such as elevation, vascularity/erythema, stiffness, collagen architecture, POSAS/VSS trajectory, or biomechanical measurements, together with etiology, anatomical tension, age, phototype, and treatment history.

A second interpretive discipline is applied throughout the review. Reduced oxidative stress is not equivalent to NRF2 pathway activation: many interventions lower ROS, 4-hydroxynonenal (4-HNE), or malondialdehyde (MDA) through direct scavenging, metal chelation, mitochondrial effects, or off-target actions on MAPK, NF-κB, TGF-β/Smad, autophagy, or ferroptosis signaling, without necessarily engaging NRF2 [24], [36], [37], [38]. We therefore use engagement-to-phenotype (E2P) as a non-ordinal attribution framework rather than a validated score or evidence hierarchy. Five domains are reported separately: (i) disease proximity/model relevance; (ii) NRF2 pathway engagement, prioritizing quantified nuclear NRF2 together with a coherent multi-gene NRF2 regulon rather than HO-1 alone [21], [22], [23], [24], [39], [40], [41], [42], [43], [44], [45]; (iii) causal NRF2 dependence; (iv) linkage to a disease-relevant scar phenotype [1], [46], [47], [48], [49], [50]; and (v) independent replication/methodological quality. For the purposes of this review, causal dependence is weighted according to the perturbation used: genetic deletion or conditional deletion with rescue provides the strongest evidence; multiple independent RNAi/CRISPRi reagents with concordant rescue provide intermediate evidence; and a single RNAi reagent or pharmacological inhibitor alone is supportive but insufficient to establish necessity [24], [51], [52]**.** The domains are non-equivalent and are not summed. Human observational studies are therefore described for disease proximity and pathway engagement without being penalized because genetic deletion is not feasible. Where NRF2 engagement or dependence is not demonstrated, effects are described as antioxidant, cytoprotective, scar-modifying, or ferroptosis-modulating rather than NRF2-dependent.

The E2P rubric is used here as a descriptive framework for organizing the evidence synthesized in this narrative review. Its categories are intended to make the basis for interpretation explicit, not to constitute a validated scoring instrument or a formally conducted systematic-review procedure. The five domains are reported separately, and evidence of NRF2 engagement is not treated as proof of causal dependence or of a scar-modifying effect. Independent duplicate extraction, formal assessment of reviewer agreement, and sensitivity analyses would be appropriate additional safeguards if the framework were subsequently implemented as a formal evidence-review method.

The review then develops the molecular architecture of the KEAP1–NRF2 redox hub (Section 2), examines the stage- and compartment-resolved redox pathobiology of HTS (Section 3), defines the redox-mechanistic circuits that drive and sustain the scar (Section 4), considers the therapeutic logic that follows from treating NRF2 as a conditional rather than uniformly protective target (Section 5), and identifies the key experiments needed to resolve NRF2 directionality in HTS (Section 6). Databases, search terms, and the targeted search supporting the negative finding in Section 3 are documented in Supplementary File S1.

## 2 The KEAP1–NRF2 axis: molecular architecture and the dual-degron logic

NRF2, encoded by *NFE2L2*, is governed less by transcriptional control than by the rate of its proteasomal degradation. Two structurally distinct degron systems contribute to this turnover. Their relative contribution may vary as a wound evolves from an acute redox-stressed environment toward a growth-factor- and mechanically loaded scar, but no study has directly measured a time-resolved transition from Neh2–KEAP1-dominant to Neh6–GSK-3β/β-TrCP-dominant turnover in the same scar-relevant fibroblast population. The temporal organization used here is therefore an inference from independently established pathway characteristics and an explicit subject for testing (Fig. 1).

### 2.1 The Neh2–KEAP1 arm: redox/electrophile sensing and the cysteine code

Under basal conditions, KEAP1 acts as a substrate adaptor for the CUL3–RBX1 E3 ubiquitin ligase, maintaining NRF2 at low abundance through continuous ubiquitination and proteasomal turnover [26], [27], [53]. KEAP1 binds the NRF2 Neh2 domain through a two-site hinge-and-latch interaction involving the high-affinity ETGE motif and the lower-affinity DLG motif, which together permit graded responsiveness to stress [54], [55]. Conformational cycling of the KEAP1–NRF2 complex adds a further layer of regulatory flexibility [56].

KEAP1 sensing is chemical and non-uniform. KEAP1 contains a constellation of reactive cysteines that respond to distinct chemical classes, forming a “cysteine code.” Within this code, Cys151 is the principal sensor for many electrophilic activators, including sulforaphane and fumarates, whereas Cys273 and Cys288 support basal repression and respond to a different chemical space [57], [58]. KEAP1 cysteines can also undergo other redox-sensitive modifications, including H_2_S-mediated persulfidation and NO-mediated S-nitrosation [24], [57], [58]. Although the relevant signaling molecules are present during cutaneous wound repair, the contribution of these modifications to NRF2 regulation in HTS has not been established.

Because different NRF2-directed agents engage different KEAP1 sensor cysteines and produce non-identical transcriptional outputs, “NRF2 activation” should not be treated as a single interchangeable action. This constraint is directly relevant to the comparison of candidate therapeutics in Section 5. Modification of KEAP1 cysteines impairs NRF2 ubiquitination, allowing newly synthesized NRF2 to accumulate, translocate to the nucleus, heterodimerize with small Maf proteins, and bind antioxidant response elements (AREs) [22], [23], [26], [27], [59]. This canonical Neh2–KEAP1 arm is the pathway most likely to be engaged by the early oxidative and electrophilic burst of the wound, including reactive aldehydes such as 4-HNE, which can act as an endogenous NRF2 activator [60]. This provides a direct point of contact with the lipid-peroxidation biology discussed in Section 4.4.

### 2.2 The Neh6–GSK-3β/β-TrCP arm and p62–KEAP1 noncanonical activation

A second, KEAP1-independent route of NRF2 turnover is governed by phosphorylation rather than cysteine chemistry, and is relevant to testing proposed NRF2 persistence in scar tissue. Glycogen synthase kinase-3β (GSK-3β) phosphorylates a serine cluster within the DSGIS motif of the NRF2 Neh6 domain, creating a phosphodegron recognized by β-TrCP within the SCFβ-TrCP/CUL1–RBX1 complex; a second, constitutive β-TrCP recognition motif, DSAPGS, contributes in parallel [61], [62], [63]. Because this route operates independently of KEAP1 and cysteine-based redox sensing, NRF2 abundance can rise or fall according to kinase signaling even when the local redox environment is not strongly altered.

The physiological logic of this pathway is that GSK-3β functions as a node in the output of growth- and nutrient-signaling pathways. Active PI3K–AKT signaling phosphorylates and inhibits GSK-3β, thereby reducing Neh6 phosphorylation and stabilizing NRF2, while AMPK may contribute under conditions of energy stress [62], [64]. An additional E3 ligase, Hrd1, can further accelerate NRF2 degradation in chronically stressed or fibrotic tissue [65]. A mechanistically distinct, still KEAP1-dependent noncanonical route operates through the selective-autophagy adaptor p62. Phosphorylation of p62 at Ser351 (mouse numbering; the corresponding human residue is Ser349) by mTORC1 increases its affinity for the KEAP1 Kelch domain by approximately one order of magnitude, allowing phosphorylated p62 to compete with NRF2 for KEAP1 and stabilize NRF2 when autophagic flux is impaired and p62 accumulates [66], [67], [68] (Section 4.3).

NRF2 therefore integrates several regulatory inputs: oxidative/electrophilic stress through the Neh2–KEAP1 arm; kinase-dependent turnover through the KEAP1-independent Neh6–GSK-3β/β-TrCP arm; and autophagy-linked phospho-p62–KEAP1 sequestration as a separate noncanonical but KEAP1-dependent route. How the contribution of these inputs changes during HTS maturation remains unknown and is treated here as a testable model rather than established disease biology.

### 2.3 The NRF2 output program: beyond antioxidant defense

The pathological interpretation of NRF2 in scar tissue depends on recognizing the breadth of its transcriptional output. Nuclear NRF2 drives genes involved in glutathione synthesis and recycling, including *GCLC*, *GCLM*, and *GSR*; detoxification enzymes such as *NQO1*; and the stress-adaptive enzyme *HMOX1/*HO-1 [22], [23], [39], [44], [45], [69], [70], [71], [72]. The biological meaning of these targets is compartment-specific. For example, HO-1 may support keratinocyte barrier cytoprotection, whereas in fibroblasts and macrophages it may influence iron handling, ferroptosis sensitivity, and inflammatory tone.

A program that remains underweighted in most scar-biology discussions is the anabolic, NADPH-generating arm of NRF2 signaling. NRF2 transcriptionally supports the pentose phosphate pathway through G6PD and PGD, as well as malic enzyme 1 (ME1) and isocitrate dehydrogenase 1 (IDH1), thereby redirecting glucose and glutamine metabolism toward biosynthesis and glutathione regeneration; this effect can be amplified under sustained PI3K–AKT signaling [73], [74]. This metabolic output couples redox defense to the biosynthetic and bioenergetic demands of proliferating, matrix-secreting cells, providing a metabolic—not merely antioxidant—rationale for why sustained NRF2 activity might support myofibroblast fitness in an established scar.

This same anabolic arm also carries a less-appreciated liability that bears directly on the persistence question. In fibroblast redox biology, oxidative stress reflects an oxidant burden that exceeds cellular buffering capacity, whereas the reductively shifted state considered here specifically refers to excessive NADPH-linked reducing pressure rather than to a generalized increase in cellular reduction state. When NADPH- and glutathione-generating output is engaged chronically and supraphysiologically, it can tip a cell from antioxidant sufficiency into an NADPH-reductive shift (an “anabolic redox shift”, distinct from the separate NADH/NAD + bioenergetic pool)—a state in which an excess of reducing equivalents, reflected in elevated NADPH/NADP^+^ and GSH/GSSG ratios, paradoxically impairs oxidative protein folding in the endoplasmic reticulum, perturbs disulfide-dependent chaperone cycles, and can itself provoke mitochondrial ROS through reductive pressure on the respiratory chain. The KEAP1–NRF2 system has been framed explicitly in terms of both oxidative and reductive stress [19]. This possibility sharpens the persistence-permissive model in two ways. First, it identifies a mechanism by which sustained Neh6-arm NRF2 activity could become maladaptive to the myofibroblast itself over long timescales, exposing an intrinsic vulnerability rather than only a survival advantage. Second, it predicts a directionally distinct and measurable signature—a reductively shifted GSH/GSSG and NADPH/NADP^+^ balance in established-scar myofibroblasts, opposite to the oxidatively shifted balance expected during early injury—a readout that could help discriminate a persistence-permissive NRF2 state from a still-compensatory one and that is directly testable with the redox biosensors described in Section 6.

NRF2 also coordinates the anti-ferroptotic axis discussed in Section 4.4 and intersects with proteostasis and mitochondrial quality control [24], [69], [73], [75]. Finally, the heme-regulated repressor Bach1 competes with NRF2 at AREs and restrains a subset of NRF2-responsive targets, including HMOX1 [39], [76]. Thus, stage- and heme-dependent Bach1 derepression may influence which NRF2 targets are actually expressed, meaning that a given level of nuclear NRF2 does not necessarily produce a uniform transcriptional output across scar stages. This dissociation between NRF2 abundance and NRF2 output is the explicit rationale for the second engagement-to-phenotype criterion defined in Section 1: because nuclear localization can be present without coordinated target induction, demonstrating a multi-gene ARE signature—rather than nuclear NRF2 alone—is required to establish that the pathway is transcriptionally engaged. The pathological consequence is that NRF2 may perform qualitatively different functions depending on which degron controls its stability and which arm of its output program is engaged—a theme developed further in Sections 3, 4.

## 3 Redox pathobiology of hypertrophic scarring across stages and compartments

Cutaneous repair proceeds through overlapping inflammatory, proliferative, and remodeling phases, each characterized by distinct cell populations, redox states, cytokine milieus, and mechanical demands. Because NRF2 converts oxidative and electrophilic stress into adaptive transcriptional programs, its pathological consequences are expected to differ across keratinocytes, fibroblasts, endothelial cells, macrophages, neutrophils, and other immune populations, as well as across the temporal arc of the lesion [28]. This section frames the stage- and compartment-resolved pathobiology that the molecular circuits in Section 4 then explain.

### 3.1 A testable stage-dependent model of NRF2 function

The dual-degron architecture described in Section 2 provides a testable temporal logic, not a deterministic stage switch. Early, maturing, established, and resolving scars overlap biologically, and chronological time should be used only as a descriptive guide alongside objective phenotype (for example elevation, vascularity/erythema, stiffness, collagen architecture, POSAS/VSS trajectory, or biomechanical measurements). Early repair is enriched for oxidative and electrophilic stress that may preferentially engage the redox-sensitive Neh2–KEAP1 arm. In this setting, NRF2 may protect against excessive oxidative injury and ROS-dependent inflammatory amplification while preserving physiological redox-dependent repair processes, including leukocyte signaling, keratinocyte migration, angiogenesis, and growth-factor signaling [22], [23], [28], [77], [78], [79]. For experimental discrimination, scar stage should be assigned using prespecified clinical and tissue features rather than elapsed time alone, and analyses should be stratified by fibroblast/myofibroblast (PDGFRα or COL1A1 with ACTA2/α-SMA), keratinocyte (KRT14), macrophage (CD68), and endothelial (PECAM1/CD31) compartments. The model makes three falsifiable predictions: (i) an early-injury, redox-responsive state will show increased nuclear-to-cytoplasmic NRF2 together with induction of a coordinated ARE panel (for example NQO1, GCLC, GCLM, and GSR) and oxidative-injury markers; (ii) a proposed established-scar persistence-permissive state will show sustained nuclear NRF2 and coordinated ARE/anti-ferroptotic output in activated fibroblasts, coupled to a persistence phenotype (ACTA2/α-SMA, COL1A1, POSTN, and contractility or matrix output) despite lower or plateaued oxidative-injury markers; and (iii) if NRF2 is instead pro-resolution in established scars, fibroblast-directed NFE2L2 loss should worsen, rather than improve, the matrix/persistence phenotype. No universal absolute nuclear/cytoplasmic NRF2 threshold has been validated for human HTS; thresholds should therefore be prespecified relative to matched normal-skin and stage-matched reference tissues, with antibody validation, blinded segmentation, batch controls, and a held-out validation set, rather than selected after outcome inspection.

An established scar, by contrast, is characterized less by acute oxidative injury than by sustained growth-factor signaling, PI3K–AKT and mTORC1 activity, and mechanical loading—the same inputs that can stabilize NRF2 through the KEAP1-independent Neh6–GSK-3β/β-TrCP arm or, separately, through phospho-p62–KEAP1 signaling (Sections 4.1, 4.3, 4.5). This predicts a qualitative shift in what nuclear NRF2 represents over the course of repair. In early injury, NRF2 activity may track and counteract local redox stress; in the established scar, it may instead track the proliferative and mechanical state of the myofibroblast and persist after the acute redox drive has subsided. Growth-signaling-maintained NRF2 would couple to the anabolic and anti-ferroptotic outputs described in Section 2.3, supporting metabolic adaptation, proteostatic buffering, glutathione-dependent defense, and ferroptosis resistance in activated fibroblasts. This provides a coherent route to persistence-permissive NRF2 activity that does not require ongoing unresolved oxidative stress [24], [29], [69], [75]. The opposing, pro-resolution interpretation remains viable; the readouts needed to distinguish these possibilities are specified in Sections 3.2, 4.

This compartmentalized model is further sharpened by fibroblast heterogeneity. Single-cell transcriptomic studies have resolved fibroblast subpopulations with distinct proliferative states and profibrotic versus remodeling identities in human hypertrophic scar and developing mouse scars [80], as well as distinct fibroblast states in keloid tissue [81]. Keloid-derived fibroblast states are considered mechanistically informative here but should not be interpreted as HTS-specific unless independently validated in HTS samples. These findings parallel cross-tissue stromal atlases that define shared pathological fibroblast phenotypes [82] and lineage-tracing studies showing that the dermis arises from functionally distinct fibroblast lineages [83]. NRF2 pathway genes, including *NFE2L2*, *KEAP1*, *NQO1*, *GCLC*, *GCLM*, and *SLC7A11*, have not yet been systematically profiled across these subpopulations in published HTS datasets. However, this heterogeneity is directly relevant: if NRF2 activity differs between myofibroblast-committed and remodeling-competent subsets, it would provide a cellular basis for the stage- and compartment-dependent consequences proposed here. Resolving this now requires more than the bulk immunostaining that has dominated the scar redox literature. Importantly, *NFE2L2* transcript abundance alone should not be interpreted as NRF2 pathway activity. The directly applicable methods are spatial and single-cell: imaging mass cytometry (e.g., Hyperion) or multiplexed antibody imaging (e.g., CODEX) can quantify nuclear NRF2 together with lineage and activation markers at single-cell resolution *in situ*, while spatial transcriptomics (Visium, MERFISH, or Xenium) can assess *NFE2L2* and *KEAP1* together with a multi-gene NRF2 regulon, including *NQO1*, *GCLC*, *GCLM*, and *SLC7A11*, across the fibroblast subclusters already defined by scRNA-seq in scar and keloid tissue [80], [81]. Interrogating NRF2 signatures across these subclusters in stage-annotated HTS biopsies is therefore both a priority and now technically feasible, and it is the foundation on which the decisive human experiment in Section 6 rests.

### 3.2 What is established in human HTS, and the central gap

Direct human HTS evidence for NRF2 activity remains limited and is rarely stage-annotated. Most available studies document oxidative-injury signatures, altered antioxidant capacity, or selected redox-associated markers rather than cell-resolved NRF2 transcriptional activity. To assess this specific gap, we performed a targeted literature audit rather than a PRISMA-compliant systematic review. PubMed/MEDLINE and Scopus were searched for the period 1 January 2000 to 31 May 2026, followed by a rapid pre-submission update on 20 June 2026 and a targeted web-accessible update check on 19 October 2026; the search scope and eligibility approach are documented in Supplementary File S1. The operational evidence sought was quantitative nuclear NRF2 immunofluorescence with cell-type co-localization in stage-annotated human HTS tissue, rather than total NRF2 abundance alone. Our targeted search did not identify an indexed study meeting these criteria. This negative finding should be interpreted as a limit of the targeted search rather than as proof that no such data exist; because the audit was not prospectively designed or registered as a systematic review, we do not present reconstructed PRISMA counts, and the search may not capture non-English-language reports, preprints, theses, or unannotated pathology-archive material. With that caveat, the available evidence establishes HTS as a redox-stressed tissue environment, but remains insufficient to determine whether NRF2 activity is deficient, compensatory but inadequate, or sustained in a manner that could support scar-cell survival [1], [16], [84]. This limited evidence base is a finding of the review rather than an artifact of selective citation, and it motivates the stage-annotated biopsy mapping proposed in Section 6.

These three possibilities are not interchangeable, and they are in principle separable by their redox-transcriptional signatures. A deficient state would show low nuclear NRF2, a weak or incoherent ARE-target panel, and persistently elevated oxidative-injury markers. A compensatory-but-inadequate state would show raised nuclear NRF2 with only partial ARE-target induction set against unresolved oxidative markers—an engaged but outmatched response. A persistence-permissive state would show sustained nuclear NRF2, a fully coordinated ARE and anti-ferroptotic panel, comparatively low oxidative-injury markers (consistent with effective redox buffering rather than ongoing stress), and a composite myofibroblast/persistence phenotype (for example α-SMA together with COL1A1/collagen I and POSTN, with COMP/COL11A1 where appropriate, plus functional matrix or contractility readouts). Making these discriminating patterns explicit converts the directional question from an interpretive dispute into a concrete measurement problem, and it defines precisely what the stage-resolved human map of Section 6 must read out.

Commonly reported readouts—including MDA, 4-HNE, the oxidative DNA-damage marker 8-hydroxy-2′-deoxyguanosine, and antioxidant proteins such as HO-1 or NQO1 [16], [85], [86]—indicate contextual redox stress or partial ARE-associated signaling, but they cannot define NRF2 directionality in the absence of nuclear NRF2 localization and coordinated target-gene induction. For example, hypertrophic scar tissue and scar fibroblasts show elevated ROS and altered antioxidant-enzyme expression, and total, rather than nuclear, NRF2 has been reported to decrease in scar fibroblasts after interventions that reduce their fibrogenic phenotype [87], [88]. Such whole-tissue or total-protein readouts are consistent with a redox-stressed environment, but they cannot localize NRF2 activity to a specific compartment or establish whether NRF2 is protective, inadequate, or persistence-permissive. A further caveat is that several sources draw on mixed scar types or primarily keloid-derived material, while whole-tissue analysis can obscure whether redox-adaptive signaling occurs in epidermal, fibroblast/myofibroblast, immune, endothelial, or appendage-associated compartments.

Direct human HTS evidence specifically resolving NRF2 signaling remains limited. The small evidence base prevents robust assessment of the consistency, inter-individual variability, and stage- or cell-type-specific direction of NRF2 activity in human scars, and it also makes independent replication difficult to evaluate. Consequently, mechanistic interpretation necessarily draws in part on primary HTS-derived fibroblasts, HTS-like animal models, mixed scar material, and non-HTS systems. These sources support biological plausibility but cannot substitute for stage-annotated, cell-resolved human HTS data or justify a directional therapeutic recommendation.

A further limitation is incomplete reporting of biological and clinical modifiers across the available human, animal, and primary-cell literature. Biological sex was not consistently reported across the reviewed human, animal, and primary-cell studies, precluding a reliable comparison of sex-dependent NRF2 effects. Other potentially influential variables, including age, hormonal status, skin phototype, scar etiology, anatomical site and mechanical tension, treatment history, and sampling stage, are variably reported and may influence both NRF2 biology and scar behavior. Where these variables are unavailable, the absence of reporting should be treated as a limitation rather than assumed to be neutral. Future human cohorts should prespecify these strata, include resolving HTS and mature normotrophic scars as comparators where feasible, and validate nuclear NRF2 antibodies with appropriate positive/negative and orthogonal controls before interpreting spatial protein measurements. The present review does not include newly collected human specimens or provide multi-center validation. To address the limited stage- and cell-resolved human evidence, future studies should recruit independent cohorts across multiple centers using harmonized tissue collection, processing, and analysis protocols. Matching or stratification criteria should be prespecified for anatomical site, age, sex, skin phototype/ethnicity, scar etiology, time since injury, treatment exposure, and mechanical tension. Spatial or single-cell profiling should quantify nuclear NRF2 in defined cellular compartments and assess its relationship to a coordinated NRF2 target-gene response and scar-associated phenotypes. To determine whether NRF2 activity contributes causally to scar persistence or resolution, these observational findings should be complemented by targeted perturbation in matched primary HTS fibroblasts or an appropriate established-scar model. Such studies are necessary to evaluate the generalizability and directionality of the proposed model; the persistence-permissive state remains a hypothesis rather than a demonstrated feature of human HTS.

### 3.3 Mechanistic insight from scar fibroblast and HTS-like models

Primary hypertrophic scar fibroblasts (HSFs), scar fibroblast cultures, and TGF-β1- or cytokine-stimulated fibroblast systems provide mechanistic access to myofibroblast differentiation, collagen I/III production, CTGF expression, contractility, ECM remodeling, NRF2 localization, ARE induction, NRF2 perturbation, ROS-linked profibrotic signaling, and ferroptosis sensitivity. However, these models do not fully reproduce the tissue architecture, immune context, vascularity, or mechanical heterogeneity of established human HTS [48], [49], [50]. In addition, some published “HTS fibroblast” datasets include keloid-derived or scar-type-unspecified cells, so conclusions from these studies should not be assumed to transfer directly to HTS without validation.

Within these models, NRF2 findings support both interpretations developed above: early or transient NRF2 activation may protect wound cells and limit redox-amplified profibrotic signaling [16], [28], whereas sustained NRF2 activity in stress-adapted states may enhance cell survival through glutathione-dependent buffering, proteostatic adaptation, and anti-ferroptotic defense [24], [33], [69], [75]. These interpretations remain model-dependent until tested in stage-annotated human tissue. Skin-context loss-of-function evidence is also informative, and one such finding deserves to be confronted directly rather than merely noted. In human dermal fibroblasts, NRF2 deficiency dysregulates matrisome gene expression and alters collagen fibrillogenesis [29]—direct loss-of-function evidence that insufficient NRF2 signaling is itself pathological for ECM organization. This does not weaken the persistence-permissive hypothesis; it disciplines it. Taken together with the proposal that sustained NRF2 may support myofibroblast survival in the established scar, it implies a non-monotonic, U-shaped relationship between NRF2 activity and healthy matrix behavior, in which both deficiency and chronic excess are maladaptive while an intermediate, appropriately timed level supports orderly repair. This non-monotonicity is exactly what the conditional-hub framing predicts and is one reason a single directional label (“antifibrotic” or “profibrotic”) fails for NRF2 in scar.

These apparently opposing observations can be reconciled without assuming that NRF2 becomes intrinsically profibrotic. The model separates the cytoprotective function of NRF2 from the net biological consequence of protecting a particular cell state. During early repair, limiting oxidative injury and inflammatory amplification may preserve reparative cells and favor resolution. In an established scar, however, the same antioxidant, metabolic, proteostatic, and anti-ferroptotic outputs could prolong the survival of already activated myofibroblasts and thereby become persistence-permissive. The proposed difference is therefore a context-dependent consequence of pathway engagement rather than a required molecular switch in NRF2 identity. Importantly, sustained NRF2 activity in established HTS could still represent a compensatory or pro-resolution response; a persistence-permissive interpretation would require stage-resolved pathway engagement together with causal evidence that fibroblast-directed NRF2 loss reduces an established-scar persistence phenotype.

Post-translational OGT/O-GlcNAcylation regulation of NRF2 has been reported in non-scar models [89], [90], but its relevance to HTS remains untested and should not be assumed to represent a KEAP1-independent mechanism. This emerging hypothesis is considered in Section 4.7. The lipid-peroxidation and ferroptosis studies that most directly probe the persistence-permissive hypothesis—including bruceine D, tubeimoside I, and emerging cell-targeted erastin delivery—are considered in Section 4.4. Across this evidence base, recurring limitations include incomplete scar-stage annotation, limited compartment resolution, and insufficient testing of NRF2 dependence (Table 1).

**Table 1:** Domain-based evidence matrix for redox-mechanistic modules linking the KEAP1–NRF2 axis to hypertrophic scar pathobiology. The E2P domains are reported separately and are not summed or ranked.

Mechanistic module	Key molecular events/proposed HTS interpretation	Disease proximity/model relevance	NRF2 pathway engagement	Causal NRF2 dependence	Scar-phenotype linkage	Independent replication/methodological quality (key refs)
Disease-proximal/directly applicable modules						
Neh2–KEAP1 arm (redox/electrophile sensing)	KEAP1–CUL3 turnover via ETGE/DLG; cysteine modification stabilizes NRF2. Proposed to be prominent during early oxidative/electrophilic injury. Proposed discriminating readouts: KEAP1 cysteine modification (if validated), nuclear:cytoplasmic NRF2, NQO1/GCLC/GCLM/GSR, and 4-HNE; stratify by phenotype-defined stage and cell compartment.	Mechanism established broadly; early-wound applicability; direct stage-specific HTS dominance not demonstrated.	Strong biochemical/mechanistic NRF2 engagement outside HTS; HTS stage-specific nuclear output unmeasured. Proposed test: quantify nuclear:cytoplasmic NRF2 and a coordinated ARE panel against matched normal-skin and stage-matched references; no universal absolute HTS threshold is validated.	No HTS test showing Neh2 dependence across scar stage.	*Protective wound/redox effects plausible; HTS-specific scar endpoint attribution limited.* Falsifiable prediction: early redox-responsive tissue shows NRF2 nuclear enrichment and coordinated ARE induction alongside oxidative-injury signals. Absence of this pattern weakens the proposed early-state model; engagement alone does not prove Neh2-specific causality.	Mechanism independently replicated; HTS temporal inference remains unvalidated [26], [27], [54], [55], [56], [57], [58], [59], [60].
Neh6–GSK-3β/β-TrCP arm (KEAP1-independent)	GSK-3β phosphorylates Neh6; β-TrCP/SCF promotes degradation; PI3K–AKT can inhibit GSK-3β and stabilize NRF2. Proposed discriminating readouts: PI3K–AKT activity; inhibitory GSK-3β phosphorylation/activity; NRF2 Neh6 phosphorylation and turnover; nuclear:cytoplasmic NRF2; ARE targets; ACTA2/α-SMA, COL1A1, POSTN, contractility and matrix output.	Core mechanism established outside HTS; PI3K–AKT and scar stiffness are disease-relevant; complete sequence not traced in HTS.	NRF2 turnover mechanism established non-HTS; stage-resolved HTS nuclear engagement not demonstrated. Test under defined matrix stiffness with time-resolved NRF2 turnover and pathway-specific AKT/GSK-3β/β-TrCP perturbation; compare nuclear NRF2 and ARE output across matched cell compartments.	No genetic/pathway-discriminating HTS test of the full stiffness→AKT→GSK-3β→Neh6 chain. Falsification/causality test: route-specific perturbation should alter NRF2 turnover and phenotype, with rescue/epistasis establishing NRF2 dependence. Failure to show route-specific effects argues against this proposed chain.	*Predicted link to activated-fibroblast stress tolerance/persistence; phenotype causality not established.* Falsifiable prediction: sustained Neh6-linked NRF2 output in established-scar activated fibroblasts associates with persistence despite lower/plateaued oxidative-injury markers; association alone is not proof of causation.	Mechanistic components replicated across systems; HTS integration remains a prediction [61], [62], [63], [64], [74].
TGF-β/Smad–NOX4–H_2_O_2_ circuit	Smad3 induces NOX4; local H_2_O_2_ reinforces profibrotic signaling and may couple PTEN–PI3K–AKT to Neh6 control.	TGF-β/NOX4 fibrosis biology supported in scar-relevant and other fibrotic systems; full NRF2 chain inferred.	NRF2 may be engaged through Neh2 redox sensing and indirectly through Neh6; direct HTS pathway mapping incomplete.	Causal support is strong for TGF-β/NOX4 fibrosis, not for the complete NRF2 linkage in HTS.	*Strong scar-relevant links to myofibroblast differentiation, collagen/CTGF and contractility.*	Core fibrosis circuit replicated; NRF2 coupling partly extrapolated [1], [20], [48], [49], [62], [74], [91], [92], [93], [94], [95], [96], [97], [98].
NRF2–NF-κB inflammatory crosstalk	Reciprocal redox-inflammatory regulation via IKK/IκB, HO-1/CO, glutathione redox control and CBP/p300 competition.	Scar inflammation is disease-relevant; detailed molecular crosstalk largely from non-HTS systems.	Pathway crosstalk supported mechanistically; HTS cell-type-resolved NRF2 engagement limited.	No HTS genetic demonstration that NRF2 is necessary for the inflammatory-scar phenotype.	*Inflammation and cytokine persistence are linked to HTS; NRF2-specific phenotype attribution remains context-dependent.*	Multiple mechanistic sources, but limited HTS-specific causal replication [16], [17], [24], [28], [99], [100], [101], [102], [103], [104], [105].
p62–KEAP1/autophagy feedback	Impaired flux can accumulate phospho-p62, which competes for KEAP1; this route is noncanonical but KEAP1-dependent. Proposed discriminating readouts: human phospho-p62 Ser349, p62–KEAP1 interaction, autophagic flux, nuclear:cytoplasmic NRF2, coordinated ARE targets, and fibroblast activation/matrix output.	Autophagy changes are reported in scar fibroblasts; the p62–KEAP1 mechanism is mainly established outside HTS.	NRF2 stabilization through phospho-p62–KEAP1 is mechanistically established; stage-specific HTS engagement unresolved. Quantify phospho-p62 and p62–KEAP1 engagement with nuclear NRF2 and ARE targets in defined cell compartments; interpret phospho-p62 abundance alongside autophagic-flux measurements.	No definitive HTS genetic p62→KEAP1→NRF2 causal chain with rescue. Causal test: perturb p62 phosphorylation or p62–KEAP1 interaction with appropriate rescue; phospho-p62 abundance alone does not establish NRF2 dependence.	*Potential links to stress tolerance and fibrotic phenotype; flux-dependent direction remains unresolved.* Falsifiable prediction: if this route contributes to HTS NRF2 stabilization, route engagement should track NRF2 output; failure of pathway-specific perturbation to alter output argues against a causal role.	Mechanism replicated outside HTS; HTS application requires flux and phospho-p62 validation [66], [67], [68], [106], [107], [108], [109], [110], [111], [112].
Iron–lipid redox and ferroptosis	Execution requires iron/PUFA-phospholipid peroxidation; defense includes SLC7A11–GSH–GPX4-associated programs plus FSP1–CoQ10, GCH1/BH4 and DHODH.	Primary HTS fibroblast and rabbit-ear evidence exists for selected pro-ferroptotic interventions; some studies are HTS-like or mixed.	Bruceine D/tubeimoside I report NRF2-axis changes; other agents show ferroptosis without demonstrated NRF2 engagement.	Genetic *NFE2L2* necessity has not been established in the key HTS studies; ML385/pharmacology is supportive only.	*Ferroptosis rescue and scar-model outcomes support a scar-relevant death phenotype; NRF2-specific attribution is narrower.*	Disease-proximal evidence is limited; bruceine D/tubeimoside I share overlapping authorship/methodology [69], [75], [113], [114], [115], [116], [117], [118], [119], [120], [121], [122], [123], [124], [125], [126], [127], [128], [129], [130], [131].
Emerging/prediction-level modules						
OGT/O-GlcNAc regulation of NRF2	Context-dependent post-translational regulation of NRF2 stability reported outside scar models; not automatically KEAP1-independent.	Non-HTS mechanistic evidence only.	No stage- or cell-resolved HTS NRF2 engagement data.	No HTS causal perturbation/rescue.	*No HTS scar-phenotype linkage.*	Hypothesis-generating; requires independent scar-specific validation [89], [90].
Mechanoredox coupling/stiffness→Neh6 prediction	Stiffness/integrin–FAK–PI3K–AKT may inhibit GSK-3β and stabilize NRF2; mechanical loading may also alter local H_2_O_2_ and ferroptosis context.	Scar stiffness/mechanotransduction is disease-relevant; stiffness→Neh6→nuclear NRF2 has not been demonstrated in HTS.	Predicted nuclear NRF2/regulon response under defined stiffness; currently unmeasured in HTS.	Requires AKT/GSK-3β/β-TrCP perturbation with redox-clamp controls; no causal HTS test yet.	Predicted effects on contractility, matrix output and persistence; no direct phenotype attribution.	Explicit testable prediction, not established pathway [11], [13], [15], [62], [74], [132], [133], [134], [135].

**Evidence reporting:** Disease proximity/model relevance, NRF2 pathway engagement, causal dependence, scar-phenotype linkage, and independent replication/methodological quality are separate, non-equivalent domains. Direct human HTS, primary HTS-derived fibroblast, HTS-like animal, keloid/mixed-scar, and non-HTS mechanistic evidence are identified explicitly.

**Disease-source labels:** “Human HTS” indicates direct human hypertrophic-scar tissue; “primary HTS fibroblast” indicates cells derived from HTS; “HTS-like” indicates animal models reproducing hypertrophic-scar features; “keloid/mixed scar” is not treated as HTS-specific; and “non-HTS mechanistic” indicates evidence used for plausibility rather than disease-specific proof.

**E2P domain framework:** The domains are not scored or summed and the framework has not been prospectively validated in HTS. A worked high-attribution example would report: quantified nuclear NRF2 with cell-type localization; a coherent multi-gene NRF2 regulon; causal *NFE2L2* deletion/conditional deletion with rescue; and a disease-relevant scar endpoint, while disease proximity and independent replication/methodological quality are reported separately. For causal dependence, multiple independent RNAi/CRISPRi reagents with concordant rescue are intermediate evidence; a single RNAi reagent or pharmacological inhibitor alone is supportive but insufficient to establish necessity.

**Abbreviations**: 4-HNE, 4-hydroxynonenal; ACSL4, acyl-CoA synthetase long-chain family member 4; ARE, antioxidant response element; CTGF, connective tissue growth factor; FAK, focal adhesion kinase; FSP1, ferroptosis suppressor protein 1; GPX4, glutathione peroxidase 4; GSH, glutathione; HSF, hypertrophic scar fibroblast; KEAP1, Kelch-like ECH-associated protein 1; LOX, lipoxygenase; LPCAT3, lysophosphatidylcholine acyltransferase 3; NOX4, NADPH oxidase 4; NRF2, nuclear factor erythroid 2-related factor 2; NSF, normal skin fibroblast; POR, cytochrome P450 oxidoreductase; PUFA, polyunsaturated fatty acid; SLC7A11/xCT, cystine/glutamate antiporter; TfR1, transferrin receptor 1; α-SMA, α-smooth muscle actin.

## 4 Redox-mechanistic circuits that drive and sustain the scar

The redox pathology of HTS is built from a small set of interacting molecular circuits in which NRF2 is embedded (Fig. 1). Before tracing these circuits, it is useful to position NRF2 alongside other redox-regulatory systems in the same tissue, because this comparison clarifies its distinctive role. NADPH oxidase 4 (NOX4) is a constitutively active, TGF-β1-inducible source of H_2_O_2_ in dermal fibroblasts and myofibroblasts that amplifies profibrotic signaling [91], [92], [93]. In this circuit, NOX4 generates ROS, whereas NRF2 coordinates transcriptional cytoprotection; scar outcome may therefore depend in part on the balance between NOX4-driven oxidant production and NRF2-driven adaptive capacity.

The thioredoxin/peroxiredoxin (TXN/PRDX) system provides rapid, catalytic, non-transcriptional clearance of H_2_O_2_ and organic hydroperoxides. Although several components of this system, including TXN1, TXNRD1, SRXN1, and PRDX1, are themselves NRF2/ARE targets [60], its peroxide-detoxifying activity is exerted enzymatically rather than through the broader transcriptional coordination of detoxification, iron handling, metabolic adaptation, and anti-ferroptotic defense. Mitochondrial SOD2/MnSOD, in turn, shapes mitochondrial redox signaling, partly in an NRF2-dependent manner, but does not directly coordinate lipid peroxidation or iron handling. NRF2 is therefore distinctive because it integrates antioxidant buffering, ferroptosis resistance, metabolic adaptation, proteostasis, and inflammatory restraint in ways that these redox systems individually do not.

### 4.1 TGF-β/Smad differentiation and the NOX4–H_2_O_2_ feed-forward loop

Fibroblast-to-myofibroblast differentiation is the defining cellular event of HTS [94]. TGF-β/Smad signaling drives α-SMA induction, contractility, collagen I/III production, CTGF expression, and ECM accumulation, thereby reinforcing scar thickness and stiffness [1], [20], [48], [49], [95]. This profibrotic program is tightly coupled to redox biology: persistent oxidant signaling, including NOX4-derived H_2_O_2_, can amplify TGF-β-linked transcriptional output and create feed-forward reinforcement between oxidative stress and matrix deposition [16], [28].

The molecular coupling to NRF2 is specific. TGF-β1, acting through Smad3, transcriptionally induces NOX4, a constitutive source of H_2_O_2_ at the endoplasmic reticulum and mitochondria [91], [92], [93]. NOX4-derived H_2_O_2_ reinforces TGF-β/Smad output, in part by oxidizing catalytic cysteines in redox-sensitive phosphatases and thereby prolonging profibrotic kinase activation. Two consequences for NRF2 follow. First, the same H_2_O_2_ can modify KEAP1 cysteines and engage the canonical Neh2–KEAP1 arm, a step demonstrated in fibroblast systems. Second, and more relevant to scar persistence, NOX4-derived H_2_O_2_ can oxidize and inactivate PTEN [96], relieving inhibition of PI3K–AKT signaling. Active AKT then inhibits GSK-3β and can stabilize NRF2 through the KEAP1-independent Neh6 arm [62], [74].

In HTS, this second route should be interpreted as an inferred mechanistic chain rather than a fully demonstrated pathway. Each step—NOX4-derived H_2_O_2_ production, PTEN oxidation, PI3K–AKT activation after PTEN loss, AKT-mediated GSK-3β inhibition, and subsequent Neh6-dependent NRF2 stabilization—has independent support, but the full sequence has not yet been traced in a single scar-fibroblast experiment. The same profibrotic ROS that amplify Smad signaling may therefore also sustain NRF2 through a growth-signaling route, allowing NRF2 to remain elevated in established scars as a consequence of the profibrotic program rather than solely as a brake on it. The 4-HNE–KEAP1 loop discussed in Section 2.1 reinforces this direction [60].

NRF2 may oppose this axis chiefly by increasing antioxidant and detoxification capacity through glutathione-axis genes, NQO1, and HO-1, thereby raising the threshold for ROS-amplified profibrotic signaling [22], [23], [24]. Direct NRF2-mediated inhibition of Smad3 phosphorylation has been reported in some non-scar fibrotic systems, including scleroderma and renal fibrosis models [97], [98], but remains unconfirmed in HTS-relevant models and should therefore be treated as a working hypothesis. The stage dependence is thus mechanistically interpretable: during early or intermediate repair, redox-driven Neh2-arm NRF2 may limit excessive fibroblast priming and TGF-β/Smad-driven differentiation, whereas in established scars, growth-signaling-driven Neh6-arm NRF2 may coexist with, and metabolically support, the very myofibroblast state it is presumed to restrain. Studies should therefore report NRF2 readouts together with α-SMA, collagen I/III, CTGF, Smad activation, NOX4 expression, and PI3K–AKT/GSK-3β status, because these measurements are needed to distinguish redox-buffering NRF2 activity from growth-signaling-driven NRF2 stabilization.

A key redox-kinetic caveat is that NOX4 predominantly generates H_2_O_2_ rather than superoxide [136], and H_2_O_2_ signaling to KEAP1 or PTEN is likely to depend on local compartmentalization because peroxiredoxins react with peroxide far more rapidly than these target cysteines [137], [138]. Thus, the proposed NOX4–PTEN–PI3K–AKT–GSK-3β–Neh6–NRF2 sequence should be tested with compartment-resolved H_2_O_2_ measurements rather than bulk ROS markers. Peroxiredoxin relay/floodgate mechanisms remain plausible but untested in HTS and are therefore treated as an emerging mechanistic extension in Section 4.7 rather than as an established component of the scar pathway.

*The prevailing direction of the fibrosis literature runs opposite to this emphasis.* This stage- and compartment-dependent reading must be weighed against the broader fibrosis evidence, which predominantly supports a protective NRF2. In most organ-fibrosis settings, NRF2 is reported to be protective: *Nfe2l2*-deficient mice develop more severe bleomycin-induced pulmonary fibrosis, with greater collagen accumulation, TGF-β expression, and lipid peroxidation [139], and KEAP1–NRF2 activation restrains fibrogenic signaling in renal and scleroderma models [97], [98]. These findings are consistent with the broadly antifibrotic activity ascribed to NRF2 activators across lung, kidney, and liver injury. The most direct challenge comes from skin itself: keratinocyte NRF2 protects against skin fibrosis by regulating epidermal injury and the inflammatory response [72]. We do not dispute this evidence; we read it as compartment-specific. Keratinocyte NRF2 acts at the epidermal interface to limit the injury and inflammatory signals that drive underlying dermal fibrosis, whereas the persistence-permissive proposal concerns sustained NRF2 within the activated dermal myofibroblast itself—a different cell, a different degron balance, and a different downstream consequence. The account advanced here is therefore not that NRF2 is globally profibrotic, but that the protective, redox-sensitive Neh2-arm function predominates during early or acute injury and in protective compartments such as the epidermis, whereas in the established, mechanically stiff scar a growth- and mechanically driven, KEAP1-independent Neh6-arm mode of NRF2 activity may instead support myofibroblast survival and anti-ferroptotic defense. On this view, the disagreement is genuine and presently unresolved. Distinguishing these directions requires the stage-resolved, cell-type-resolved, and genetic-dependence testing outlined in Section 6, rather than a presumption either way. The strongest caveat remains that the disease-proximal evidence for the persistence-permissive direction is narrow and partly single-group (Section 4.4), so the protective interpretation cannot yet be excluded for human HTS.

### 4.2 NF-κB and inflammatory persistence

Persistent inflammation is a central driver of HTS because it sustains cytokine loops, fibroblast activation, and pathological remodeling [99]. IL-6, TNF-α, and TGF-β reinforce myofibroblast persistence and matrix deposition, while immune-cell-derived ROS amplify localized oxidative stress and NF-κB activation, creating a feed-forward fibroinflammatory niche [16], [17], [28], [100]. In this setting, NRF2 and NF-κB form a reciprocal redox-inflammatory circuit rather than two independent pathways. NRF2 can attenuate ROS-dependent NF-κB amplification by limiting IκB degradation and IκB kinase (IKK) activity, partly through KEAP1-linked modulation of IKK signaling and through glutathione-dependent redox control of IKK cysteine residues, by HO-1/carbon-monoxide (CO)-mediated inflammatory restraint, and by competing for the shared co-activator CBP/p300, to which NRF2 binds through two cooperative Neh domains [101], although the magnitude of this effect *in vivo* remains context-dependent and debated [17], [24], [102]. Conversely, activated NF-κB p65 can suppress ARE-driven transcription by recruiting a corepressor complex that displaces CBP from NRF2 and promotes HDAC3 recruitment to MafK [103]. This provides a plausible mechanism by which a chronically inflamed scar environment could blunt NRF2 transcriptional output even when oxidative stress is present. Inflammatory persistence itself is well supported in HTS, whereas the detailed molecular NRF2–NF-κB crosstalk described above is derived largely from non-HTS systems and should therefore be regarded as mechanistic extrapolation rather than HTS-specific proof.

The pathological consequence of this crosstalk is likely compartment-specific. In macrophages, NRF2 activity may favor inflammatory resolution by modulating polarization and repressing proinflammatory cytokine genes such as IL-6 and IL-1β through mechanisms reported to be partly independent of redox status, with additional links to resolution-phase prostaglandin signaling [104], [105]. In fibroblasts, however, NRF2 activity may have a different meaning: it may alter stress tolerance, cytokine responsiveness, metabolic fitness, and survival within the inflammatory niche [28]. Thus, the same NRF2 signal could contribute to resolution in immune compartments while supporting persistence in stress-adapted fibroblast populations. This distinction is critical for HTS, where TGF-β/Smad-driven differentiation and NF-κB-linked inflammatory persistence remain the dominant and best-evidenced pathogenic circuits. The more detailed treatment of autophagy and ferroptosis below should therefore be read as an emerging mechanistic extension of these core fibroinflammatory pathways, not as a claim that they supersede them.

### 4.3 p62–autophagy and noncanonical p62–KEAP1 activation

Autophagy maintains proteostasis and organelle quality control, including mitophagy, thereby limiting ROS generation from damaged mitochondria. In hypertrophic scar fibroblasts, autophagy-related LC3 activity has itself been linked to the fibrotic phenotype [106]. The mechanistic bridge to NRF2 is the phospho-p62 pathway described in Section 2.2. mTORC1-dependent phosphorylation of p62 at Ser351 (mouse; Ser349 in human) markedly increases its affinity for the KEAP1 Kelch domain, allowing phosphorylated p62 to compete with NRF2 for KEAP1 and stabilize NRF2 through a noncanonical p62–KEAP1 route when autophagic degradation is impaired and p62 accumulates [66], [67], [68], [107]. In this setting, p62 functions not only as an autophagy adaptor but also as a stress-responsive signaling hub. NRF2, in turn, transcriptionally induces *SQSTM1* and broader macroautophagy genes, creating a feed-forward loop that can reinforce stress adaptation [108], [109], [110]. The phospho-p62–KEAP1 mechanism is established primarily in non-scar systems; its stage-specific engagement and causal contribution to NRF2 activity in HTS fibroblasts remain unproven. In HTS, fibroblasts are exposed to sustained oxidative stress, relative hypoxia, nutrient stress, inflammatory cytokines, and matrix stiffness—conditions that increase proteostatic and mitochondrial quality-control demand and can activate mTORC1 in stiff, growth-factor-rich niches. Two biologically distinct outcomes must therefore be separated. Efficient autophagy may be reparative, clearing damaged organelles, reducing mitochondrial ROS, and supporting scar resolution. By contrast, impaired or incomplete autophagic flux may permit accumulation of phosphorylated p62, drive noncanonical p62–KEAP1 NRF2 activation, and engage stress-tolerance programs that support activated-fibroblast persistence [66], [67], [108]. The direction of this pathway is therefore not intrinsically protective or pathogenic; it depends on whether autophagy is complete and adaptive or blocked and persistence-promoting, and on the growth-signaling state of the scar fibroblast. This distinction has an important methodological consequence. Elevated steady-state p62 or LC3-II cannot by itself distinguish increased autophagosome formation from impaired autophagic degradation, and the latter is precisely the condition expected to sustain p62-KEAP1 noncanonical NRF2 signaling. The phosphorylated p62 pool (Ser351/Ser349) is the specific species that competes with NRF2 for KEAP1, so it—rather than total p62—is the readout that links autophagic status to noncanonical NRF2 activation. HTS studies should therefore measure LC3-II and p62 turnover with and without lysosomal inhibition, assess p62 phosphorylation at this site where feasible, and link p62–KEAP1–NRF2 activity to disease-relevant endpoints such as α-SMA expression, collagen output, apoptosis resistance, scar elevation, and *in vivo* remodeling [111], [112].

### 4.4 Iron–lipid redox and ferroptosis

Ferroptosis is a regulated form of cell death driven by iron-dependent lipid peroxidation [113]. It occurs when lipid hydroperoxides accumulate beyond the detoxifying capacity of antioxidant systems, particularly under conditions of impaired cystine uptake, reduced glutathione synthesis, or insufficient GPX4 activity [75], [114]. Its relevance to HTS depends on separating two linked but distinct processes: the execution machinery, which generates oxidizable lipid substrates and propagates lipid peroxidation, and the defense machinery, which detoxifies lipid hydroperoxides and limits iron-dependent propagation. This distinction is central because NRF2 primarily governs the defensive side of the pathway and is therefore mechanistically positioned to protect activated fibroblasts from ferroptotic clearance.

Execution depends on both membrane lipid composition and iron availability. Acyl-CoA synthetase long-chain family member 4 (ACSL4) and lysophosphatidylcholine acyltransferase 3 (LPCAT3) esterify long-chain ω6 polyunsaturated fatty acids, principally arachidonic and adrenic acid, into membrane phospholipids, thereby generating the obligatory substrate for lethal lipid peroxidation. ACSL4 is the principal determinant of ferroptosis susceptibility and is also a practical biomarker of ferroptosis-prone cells [115], [116]. Lipid peroxidation can then be initiated enzymatically by lipoxygenases and cytochrome P450 oxidoreductase, and propagated non-enzymatically by labile iron through Fenton chemistry. This distinction is important for interpreting scar-fibroblast datasets: ACSL1, reported as induced in the tubeimoside I scar-fibroblast dataset of Table 2, is mechanistically distinct from ACSL4, and future studies should confirm whether ACSL4, the more directly informative ferroptosis-sensitivity isoform, is altered in HTS-relevant systems.

**Table 2:** Domain-based attribution matrix for NRF2-directed and ferroptosis-modulating interventions in HTS and scar-relevant cutaneous fibrosis.

Intervention/class	Mechanism/claim	Disease proximity/model relevance	NRF2 pathway engagement	Causal NRF2 dependence	Scar-phenotype linkage	Independent replication/methodological quality	Refs
Group A — NRF2 activators (benchmark pharmacology; no controlled HTS efficacy data)							
Sulforaphane	Electrophilic KEAP1 modifier; benchmark NRF2 activator.	Skin-context/non-HTS; no controlled HTS efficacy study.	NRF2 nuclear/ARE engagement established in other contexts.	No HTS genetic causal test.	No controlled HTS scar endpoint.	Replicated NRF2 pharmacology; HTS efficacy untested.	[24], [140], [141], [142], [143]
Dimethyl fumarate	Electrophilic KEAP1 modification plus NRF2-independent succination/metabolic effects.	Clinical non-HTS inflammatory disease; fibrosis evidence non-HTS.	NRF2 engagement established, but not exclusive.	Pharmacology does not establish NRF2 necessity in HTS.	No controlled HTS scar endpoint.	Well-characterized agent; disease extrapolation required.	[37], [144], [145]
CDDO derivatives/RTA 408	Potent electrophilic KEAP1 modification; strong NRF2 activation.	Non-HTS wound/skin and systemic clinical experience.	Strong pathway engagement outside HTS.	No HTS causal-dependence test.	No controlled HTS scar efficacy; systemic safety liabilities relevant.	Clinical safety/engagement data exist outside HTS.	[24], [146], [147], [148], [149]
Group B — NRF2 suppressors/destabilizers (disease-proximal evidence)							
Bruceine D	PIM1 degradation with NRF2 destabilization; ferroptosis-associated changes.	Primary HTS fibroblasts + rabbit-ear; study also framed across broader skin fibrosis.	Nuclear/total NRF2 and downstream changes reported.	No genetic *NFE2L2* deletion/rescue; pharmacological mechanism only.	Ferroptosis-related and scar-model endpoints improved.	*Single research group; overlaps in provenance/methodology with tubeimoside I study.*	[126]
Tubeimoside I	PI3K–AKT suppression with plausible restoration of GSK-3β/β-TrCP-mediated NRF2 turnover; ML385 phenocopy.	Primary HTS fibroblasts + rabbit-ear.	NRF2-axis suppression reported.	ML385 is supportive but insufficient to establish necessity; no genetic rescue.	Pro-ferroptotic and scar-model endpoints improved.	*Single/overlapping research-group evidence; independent replication needed.*	[62], [63], [64], [127]
Group C — Ferroptosis-modulating agents; inclusion does not imply NRF2 mediation							
FAP-targeted sEV-erastin microneedle	Cell-directed system x_c^−^ inhibition/GSH depletion; ferroptosis rescue shown.	Primary HSF/HDF selectivity + rabbit-ear HTS-like model.	NRF2-axis transcripts reported, but nuclear NRF2/regulon engagement not established.	No genetic NRF2 dependence test.	Ferrostatin-1 rescue plus scar elevation/collagen improvement.	*Strong ferroptosis attribution; NRF2 attribution remains untested.*	[128]
Dioscin	Mitochondrial oxidative stress and lipid peroxidation.	Scar-model system/HTS-like.	NRF2 engagement not demonstrated.	NRF2 dependence untested.	Ferroptosis component supported by rescue; apoptosis also occurs.	*Interpret as ferroptosis-modulating, not NRF2-mediated.*	[129]
Hypericin photodynamic therapy	Photoactivated oxidant generation and lipid peroxidation.	Scar-model system/HTS-like.	NRF2 engagement not demonstrated.	NRF2 dependence untested.	Ferroptosis rescue reported with co-occurring apoptosis.	*Interpret as ferroptosis-modulating, not NRF2-mediated.*	[130]
Fraxinellone	Lysosomal cathepsin-B leakage/SAT1-linked lipid-redox stress.	Scar-model system/rabbit-ear HTS-like.	NRF2 engagement not demonstrated.	NRF2 dependence untested.	Ferrostatin-1-rescued ferroptosis and *in vivo* scar effects.	*Interpret as ferroptosis-modulating, not NRF2-mediated.*	[131]
Group D — NRF2-dependence tool compounds							
Brusatol; ML385	Mechanistic inhibitor tools; brusatol is nonspecific and ML385 remains pharmacological.	Used across mechanistic systems; ML385 in tubeimoside I context.	Can suppress NRF2-associated readouts.	Single pharmacological inhibitor alone is insufficient; require orthogonal genetic validation.	Tool-dependent phenotype only.	*Interpret with off-target/incomplete-dependence caution.*	[41], [42]
Group E − Local-delivery framework examples; primary mechanism is not NRF2							
Losartan-loaded microneedle	AT1R blockade; local-delivery example, not primarily NRF2.	HTS-like scar-targeted platform.	Not established/not primary mechanism.	Not applicable to NRF2 causality.	Local antifibrotic scar endpoint.	*Delivery example only.*	[150]
Asiaticoside/5-FU microneedle	TGF-β/Smad modulation + antiproliferative 5-FU; not primarily NRF2.	HTS-like delivery model; 5-FU has clinical scar use.	Not established/not primary mechanism.	Not applicable to NRF2 causality.	Local scar/myofibroblast/ECM endpoints.	*Delivery framework; NRF2 attribution not implied.*	[151], [152], [153], [154]

**Grouping:** Interventions are organized by mechanism class, but attribution is reported through separate E2P domains rather than a score. Pharmacological pathway modulation is not treated as equivalent to genetic gain- or loss-of-function.

**Interpretation:** Direction of NRF2 or ferroptosis change is described only where directly measured; pathway engagement and causal dependence are separate domains.

**Disease-source:** labels follow Table 1 and distinguish direct HTS, primary HTS-derived fibroblast, HTS-like animal, mixed/keloid, and non-HTS mechanistic evidence.

**E2P domain framework:** Domains are not summed. A worked high-attribution profile would include quantified nuclear NRF2 plus a coherent multi-gene regulon, causal *NFE2L2* deletion/conditional deletion with rescue, and a disease-relevant scar endpoint, with disease proximity and independent replication/methodological quality reported separately. Genetic deletion/rescue is stronger causal evidence than multiple independent RNAi/CRISPRi reagents, which are stronger than a single RNAi reagent or pharmacological inhibitor. Dioscin, hypericin, and fraxinellone are included as ferroptosis-modulating interventions; their inclusion does not imply NRF2 mediation.

Abbreviations: ARE, antioxidant response element; CDDO, 2-cyano-3,12-dioxooleana-1,9-dien-28-oic acid; FPN1, ferroportin-1; 5-FU, 5-fluorouracil; GPX4, glutathione peroxidase 4; HO-1, heme oxygenase-1; HSF, hypertrophic scar fibroblast; KEAP1, Kelch-like ECH-associated protein 1; NRF2, nuclear factor erythroid 2-related factor 2; NSF, normal skin fibroblast; PIM1, proviral integration site for Moloney murine leukemia virus 1 kinase; SLC7A11/xCT, cystine/glutamate antiporter; SSc, systemic sclerosis.

On the defensive side, NRF2 functions not as a regulator of a single anti-ferroptotic gene, but as a coordinated transcriptional hub for multi-layered ferroptosis resistance. *SLC7A11*/xCT and glutathione-synthesis genes such as *GCLC* and *GCLM* contribute to cystine import and glutathione supply within NRF2-coordinated defense programs, while the NADPH-generating enzymes discussed in Section 2.3 support glutathione regeneration and GPX4 function. NRF2 is also associated with iron-buffering and iron-export programs involving *FTH1*, *FTL*, and *SLC40A1*/ferroportin, thereby limiting the labile iron pool available for lipid-peroxidation propagation [69], [117], [118], [119]. This NRF2-dependent defense intersects with glutathione-independent ferroptosis control through ferroptosis suppressor protein 1 (FSP1) and CoQ10, which detoxify lipid peroxyl radicals in parallel with GPX4 [120], [121], while iron availability is further shaped by NCOA4-mediated ferritinophagy [122]. The GCH1/BH4 axis and mitochondrial DHODH represent additional parallel ferroptosis-defense systems whose relevance to HTS remains unestablished [123], [124]. GPX4 protein abundance is also constrained by selenocysteine incorporation and the mevalonate–isopentenyl-pyrophosphate pathway that supplies selenocysteine-tRNA, a layer not directly controlled by NRF2. Thus, NRF2 primarily governs GPX4 substrate and cofactor supply, redox regeneration, and iron buffering rather than GPX4 abundance per se. The glutathione-independent arm is not merely measurable but therapeutically consequential. If myofibroblast ferroptosis resistance is sustained even partly through FSP1–CoQ10 (or the related DHODH-dependent defense at the mitochondrial inner membrane) rather than the SLC7A11–GSH–GPX4 axis alone, then NRF2-axis suppression on its own would be insufficient to sensitize these cells, and effective pro-ferroptotic therapy would require parallel inhibition of the glutathione-independent arm. This is a directly testable and clinically consequential prediction, and it identifies FSP1/CoQ10, the GCH1/BH4 tetrahydrobiopterin axis, and DHODH as priority readouts in any scar-fibroblast ferroptosis study. The key point is that sustained NRF2 activity can simultaneously increase cystine uptake, glutathione synthesis, NADPH-dependent glutathione regeneration, lipid-peroxide detoxification capacity, and iron sequestration. In established HTS, this coordinated defense program could provide activated fibroblasts with multi-layered ferroptosis resistance rather than merely incremental antioxidant protection.

During early repair, NRF2-driven ferroptosis resistance may be beneficial by limiting lipid-peroxidation-amplified inflammation and excessive cell loss, thereby helping to preserve tissue integrity. In established HTS, however, the same SLC7A11/xCT–GSH–GPX4 defense program, reinforced by NADPH supply and iron sequestration, could protect activated fibroblasts and myofibroblasts from ferroptotic clearance and thereby support scar persistence. The lipid aldehyde 4-HNE illustrates its possible dual role as both a product and marker of lipid peroxidation and a bioactive electrophile capable of modifying signaling proteins, including KEAP1, and influencing inflammatory/profibrotic pathways. As discussed in Section 2.1, sublethal 4-HNE may activate NRF2 through KEAP1 modification and promote detoxification, but whether sustained 4-HNE–KEAP1–NRF2 feedback contributes to fibroblast persistence in HTS remains a testable hypothesis rather than an established mechanism [60]. Markers such as 4-HNE and MDA indicate lipid-redox stress but do not by themselves prove ferroptosis unless accompanied by validated ferroptosis-specific readouts and mechanism-specific rescue [85], [125]. Accordingly, claims of ferroptosis targeting should require evidence of lipid-peroxidation accumulation, altered ferroptosis-defense systems such as GPX4 and SLC7A11/xCT, ideally ACSL4 or iron-related readouts, and rescue by ferroptosis inhibitors or iron chelation [33], [66], [69].

Within this framework, bruceine D and tubeimoside I remain the most directly relevant NRF2-linked HTS examples, because both studies used primary human HTS-derived scar fibroblasts compared with normal skin fibroblasts. Bruceine D promotes degradation of PIM1 kinase, destabilizes NRF2, lowers GPX4, SLC7A11/xCT, HO-1, and FPN1, and increases lipid peroxidation, thereby acting as an indirect NRF2 down-regulator [126]. Tubeimoside I suppresses PI3K–AKT signaling upstream of NRF2. Because PI3K–AKT inhibition is expected to restore GSK-3β activity and promote β-TrCP-mediated NRF2 degradation through the Neh6 degron [62], [63], this provides a plausible molecular link between PI3K–AKT suppression and NRF2 loss. It also explains why ML385-mediated NRF2 inhibition phenocopies, rather than opposes, the antifibrotic and pro-ferroptotic effects of tubeimoside I [127]. However, parallel NRF2-independent effects of PI3K–AKT suppression cannot be excluded without genetic validation.

A recent FAP-targeted small extracellular vesicle microneedle system delivering erastin refines but does not settle the model. This approach selectively induced ferroptosis in hypertrophic scar fibroblasts and improved rabbit-ear scar outcomes, strengthening the rationale for local, cell-directed ferroptosis sensitization, but it did not establish NRF2 engagement [128]. By contrast, dioscin-induced mitochondrial oxidative stress, hypericin-mediated photodynamic therapy, and fraxinellone-mediated lysosomal cathepsin B leakage each report ferroptosis alongside apoptosis or another co-occurring death program in scar fibroblasts. In each case, mechanism-specific rescue supports a genuine ferroptosis-dependent component: dioscin-induced lipid peroxidation, iron accumulation, and antioxidant-protein loss are reversed by ferrostatin-1 and the ROS scavenger NAC [129]; hypericin-induced ROS, lipid peroxidation, and iron accumulation are reversed by ferroptosis inhibitors, with HMOX1 implicated as the effector [130]; and fraxinellone-induced ferroptosis, mediated through cathepsin-B/SAT1-driven lipid peroxidation, is blocked by ferrostatin-1 in both cultured fibroblasts and the rabbit-ear scar model [131]. None of these three studies demonstrates NRF2 engagement, so they are best interpreted as ferroptosis-targeting tools rather than evidence for the NRF2 axis.

Taken together, ferroptosis in HTS should be viewed as the net result of an iron- and PUFA-phospholipid-dependent execution arm opposed by multiple ferroptosis-defense systems, rather than as a process controlled by NRF2 alone. Mechanistic studies should therefore evaluate labile iron, ACSL4/LPCAT3-dependent PUFA-phospholipid remodeling, and lipid peroxidation together with the SLC7A11–GSH–GPX4 axis and parallel FSP1–CoQ10, GCH1/BH4, and DHODH defenses. Accordingly, 4-HNE or MDA alone should be interpreted as markers of lipid-redox stress rather than proof of ferroptosis, which requires mechanism-specific rescue and appropriate cell-death controls. Within this framework, dioscin, hypericin, and fraxinellone are best considered ferroptosis-modulating interventions unless NRF2 dependence is demonstrated directly.

Importantly, the available evidence is substantially asymmetric between the two directional hypotheses. The protective-NRF2 direction is supported by germline and conditional NRF2 loss-of-function *in vivo* across at least three organ systems—lung [139], kidney and scleroderma-type fibrosis [97], [98], and skin, including human dermal fibroblast loss-of-function affecting the matrisome [29] and keratinocyte-restricted protection against skin fibrosis [72]—spanning genetic and pharmacological designs from multiple independent groups. Taken together, the broader fibrosis literature therefore provides substantially stronger causal support for a protective or antifibrotic role of NRF2 than the current evidence provides for a persistence-permissive role in HTS. The latter should consequently be regarded as a disease-proximal but comparatively weak hypothesis until it is independently replicated and supported by genetic NRF2 perturbation in established HTS. Disease-proximal evidence also includes an independent hypoxia study in human HTS fibroblasts: Nrf2 overexpression increased, and Nrf2 knockdown reduced, HIF-1α/BNIP3-associated mitophagy and fibroblast viability, migration, and invasion [35]. This provides gain-/loss-of-function evidence that NRF2 can influence HTS fibroblast behavior *in vitro*, but it does not establish stage-resolved nuclear NRF2 in human scar tissue or prove that sustained NRF2 is persistence-permissive in established scars. The proposed persistence-permissive direction is instead supported primarily by the bruceine D study [126] and the tubeimoside I study [127] in HTS-derived fibroblasts and rabbit-ear scar. These studies share overlapping authorship and similar experimental methodology, and neither establishes NRF2 necessity by genetic *NFE2L2* ablation; the tubeimoside I study relies on ML385, which we have argued is insufficient on its own (Sections 1, 5.2). Two further limitations compound this. First, the bruceine D study is framed as a broader skin pan-fibrosis investigation spanning hypertrophic scar, keloid, and systemic sclerosis, so its HTS-specific component should not be over-weighted. Second, the FAP-sEV erastin scar study and the non-scar OGT–O-GlcNAcylation mechanistic report [89], [128], while consistent respectively with local ferroptosis sensitization in scar fibroblasts and a post-translational route to NRF2 stabilization, were not designed to test NRF2 directionality in HTS. We therefore treat the persistence-permissive model as the less-supported of two live hypotheses and pre-specify the result that would move it from hypothesis to supported claim: fibroblast-restricted NRF2 deletion imposed on an established scar—rather than during early injury—should reduce a composite persistence phenotype including α-SMA together with matrix/activation markers such as COL1A1/collagen I and POSTN, plus collagen organization, scar-elevation index, and stiffness (Section 6). Absent that result, the protective interpretation cannot be excluded for human HTS. The persistence-permissive hypothesis thus has its strongest model-level support in this section, but that support remains narrow, partly single-group, and not yet validated in stage-annotated human tissue.

### 4.5 Mechanoredox coupling and a predicted Neh6-arm route to NRF2 stabilization

Mechanical loading and matrix stiffening are major determinants of HTS persistence. Myofibroblast contractility can itself liberate active TGF-β1 from latent ECM stores, directly coupling tissue stiffness to profibrotic signaling [132]. The connection to NRF2 is similarly specific and is central to the persistence-permissive model, because mechanical signaling could sustain NRF2 independently of ongoing oxidative stress.

Mechanical force generates redox signals that feed transcriptional programs. Stiffness and tension are transduced through integrin–focal adhesion kinase (FAK)–RhoA/ROCK signaling and the actomyosin cytoskeleton. These pathways can raise intracellular ROS through increased mitochondrial ROS under cytoskeletal tension and through assembly or activation of NADPH oxidases, including NOX4, at focal adhesions. Mechanosensitive Piezo-family channels further couple membrane tension to calcium influx, which can stimulate both NOX activity and mitochondrial ROS generation [133]. The localization of NOX4 to focal adhesions is mechanistically significant in light of the redox-kinetic argument in Section 4.1: by concentrating H_2_O_2_ production within a defined adhesion nanodomain, mechanical loading provides exactly the spatially restricted, locally saturating peroxide source that could oxidize PTEN and KEAP1 despite bulk peroxiredoxin buffering. Mechanical state and redox state should therefore be viewed as an integrated mechanoredox circuit rather than as parallel inputs.

YAP and TAZ provide an additional link between mechanics, redox regulation, and ferroptosis sensitivity. These principal stiffness-sensing effectors are activated by cytoskeletal tension and translocate to the nucleus on stiff matrices [134]. YAP/TAZ activity is redox-influenced, and YAP has been reported to promote ferroptosis sensitivity by upregulating ACSL4 and the transferrin receptor [135]. This could establish the lipid-substrate and iron context on which the NRF2-dependent ferroptosis-defense program described in Section 4.4 acts. Whether this YAP/TAZ–ferroptosis axis operates in scar fibroblasts remains untested. Nevertheless, it raises an important possibility: a stiff scar may simultaneously increase ferroptosis-execution capacity through YAP/TAZ-linked ACSL4 and transferrin-receptor expression, while also increasing NRF2-dependent defense. The balance between these opposing arms may help determine whether activated myofibroblasts are eliminated or persist.

Stiffness may also provide a direct route to KEAP1-independent Neh6-mediated NRF2 stabilization. The integrin–FAK pathway that activates YAP/TAZ can also activate PI3K–AKT signaling. Active AKT inhibits GSK-3β, which would reduce Neh6 phosphodegron activity and stabilize NRF2 independently of KEAP1 and cysteine-based redox sensing [62], [74]. This chain remains inferred in scar fibroblasts: integrin–FAK–PI3K–AKT–GSK-3β signaling and Neh6-arm NRF2 stabilization are each supported in their respective biological contexts, but no published study has yet measured nuclear NRF2 as a function of defined substrate stiffness in scar-derived fibroblasts. The model therefore yields a concrete and falsifiable prediction that unifies Sections 2, 4.1, 4.5: increasing substrate stiffness should elevate nuclear NRF2 in scar fibroblasts through FAK–PI3K–AKT–GSK-3β signaling, even without a proportional increase in oxidative stress, whereas matrix softening or inhibition of FAK or AKT should reduce nuclear NRF2. Critically, a simple positive correlation between stiffness and nuclear NRF2 would not by itself establish the mechano-route, because mechanical loading also raises ROS and could therefore stabilize NRF2 through the redox-sensitive Neh2 arm. A useful operational threshold is not a universal numerical ROS or strain cutoff, which current HTS data cannot justify, but the experimentally observed inflection at which graded scar-relevant stiffness or cyclic strain changes nuclear NRF2/ARE output relative to local H_2_O_2_. The discriminating experiment must show that stiffness-dependent nuclear NRF2 (i) persists under an antioxidant clamp—for example, in the presence of N-acetylcysteine or when a compartment-targeted H_2_O_2_ biosensor confirms no rise in local peroxide—and (ii) is abolished by AKT or GSK-3β manipulation but not by KEAP1 knockdown. Only that combination isolates the KEAP1-independent Neh6 mechano-route from residual Neh2/redox activation.

If confirmed, NRF2 activity in established HTS would represent, at least in part, a mechano-transcriptional output sustained by the stiff scar niche itself. This would provide a mechanism for persistence-permissive NRF2 activity that does not require ongoing unresolved oxidative stress and that can be tested directly using stiffness-tunable substrates. Future mechanoredox studies should therefore pair NRF2 readouts, including nuclear localization and a multi-target ARE signature, with substrate stiffness, collagen alignment, α-SMA expression, contractility, YAP/TAZ localization [134], Piezo1 activity, FAK–AKT–GSK-3β status, and scar biomechanics. These measurements are needed to determine whether NRF2 engagement actually modifies the mechanically reinforced scar niche rather than merely lowering oxidative markers.

Together, the autophagy–p62, ferroptosis, and mechanoredox circuits illustrate how multiple pathways involve NRF2 in activated fibroblasts, but they do not establish a single KEAP1-independent late-scar program. Whether this activity promotes scar resolution or permits scar persistence likely depends on scar stage, cellular compartment, mechanical environment, and duration of pathway engagement. Because the mechanistic density of Section 4 could otherwise obscure the distinction between established evidence and mechanistic inference, it is useful to state explicitly what is established and what remains unresolved. Several elements are supported in scar-relevant or mechanistically applicable systems: TGF-β/Smad-driven myofibroblast differentiation and its reinforcement by NOX4-derived H_2_O_2_ (Section 4.1); NF-κB-linked inflammatory persistence and reciprocal NRF2–NF-κB crosstalk (Section 4.2); the noncanonical phospho-p62–KEAP1 route to NRF2 stabilization, demonstrated primarily in non-scar systems (Section 4.3); NRF2's coordinated transcriptional control of anti-ferroptotic defense (Section 4.4); and, in two largely single-group studies, pharmacological NRF2 suppression that sensitizes HTS-derived fibroblasts to ferroptosis and reduces scar-model endpoints (Section 4.4). By contrast, several questions remain unresolved and define the experimental agenda of Section 6: whether nuclear NRF2 in human myofibroblasts rises, falls, or redistributes as scars mature; whether NOX4-derived H_2_O_2_ oxidizes PTEN in scar fibroblasts, thereby activating PI3K–AKT signaling, inhibiting GSK-3β, and stabilizing NRF2 through the Neh6 degron; whether matrix stiffness stabilizes NRF2 in the absence of overt redox stress, as predicted in Section 4.5; and whether the persistence-permissive direction extends beyond bruceine D and tubeimoside I. The molecular events, their consequences for scar pathology, and the strength of disease-specific evidence are summarized in Table 1, which reports disease proximity/model relevance, NRF2 engagement, causal dependence, scar-phenotype linkage, and independent replication/methodological quality separately rather than as a single rank. The directional relationships among NOX4, NF-κB, p62, ferroptosis, and mechanoredox signaling remain unresolved in HTS and should not be inferred from co-occurrence alone. A discriminating design would combine time-resolved, compartment-specific redox reporters with factorial perturbation of NOX4/ROS, RELA/NF-κB, SQSTM1/p62–KEAP1, and ferroptosis-defense nodes, followed by NFE2L2 loss/rescue and scar-relevant functional endpoints. The predicted order of events should be specified in advance, and mediation should be supported by reversal/rescue rather than correlation. Until such stage-specific causal evidence is available, local NRF2 modulation is a research hypothesis and should not be described as an established therapeutic recommendation.

### 4.6 Paracrine redox signaling in the scar niche

The intracellular KEAP1–NRF2 circuitry above operates within a scar niche that is also shaped by intercellular, paracrine redox communication. Fibroblast-, keratinocyte-, macrophage-, and endothelial-derived soluble factors and small extracellular vesicles (sEVs) can carry profibrotic, inflammatory, mitochondrial, and redox cargo between cell types in the scar, and could therefore alter NRF2 activity indirectly in recipient cells even without a direct change in that cell's own oxidative or electrophilic exposure [155], [156]. This intercellular layer is mechanistically distinct from, and less well characterized than, the intracellular KEAP1–NRF2 mechanisms described in Section 2: direct evidence that HTS-derived sEVs regulate NRF2 in a stage-specific manner is still limited, and the FAP-targeted sEV-erastin system discussed in Sections 4.4, 5.3 is a delivery vehicle rather than evidence of endogenous paracrine NRF2 control [128]. Paired donor/recipient-cell experiments—sEV isolation and characterization, uptake tracing, recipient-cell nuclear NRF2 localization, ARE-regulon readouts, and genetic *NFE2L2* perturbation in the recipient cell—would help establish whether paracrine sEV signaling is a meaningful route to NRF2 modulation in HTS, rather than an incidental correlate of the fibroinflammatory niche.

### 4.7 Emerging hypotheses and testable extensions

Several secondary mechanisms are mechanistically plausible but presently too weakly supported in HTS to carry the central model. These include peroxiredoxin redox-relay or local ‘floodgate’ control of NOX4-derived H_2_O_2_ signaling [136], [137], [138], context-dependent OGT/O-GlcNAc regulation of NRF2 stability [89], [90], and the relative contribution of parallel ferroptosis defenses such as FSP1–CoQ10 [120], [121], GCH1/BH4 [123], and DHODH [124]. These mechanisms should be treated as extensions to be tested with compartment-resolved redox sensors, pathway-specific perturbation, and stage-annotated scar models rather than as demonstrated HTS pathways. Their inclusion is intended to define experiments that could refine or falsify the broader model, not to increase the apparent evidence for persistence-permissive NRF2.

## 5 Therapeutic implications: targeting a conditional redox hub

Because NRF2 governs redox tone, inflammatory restraint, stress adaptation, proteostasis, metabolic buffering, and lipid-peroxidation control, it represents an attractive but potentially double-edged therapeutic target in HTS [22], [23], [26], [27]. The mechanistic framework developed above argues that NRF2-directed therapy should not be conceived as uniformly activating or inhibiting the pathway. Rather, intervention direction should remain conditional on scar phenotype, cellular compartment, and demonstrated pathway engagement. Early, redox/electrophile exposure may engage the Neh2–KEAP1 arm, whereas growth-factor and mechanical inputs in established scars could increase the contribution of the Neh6–GSK-3β/β-TrCP arm; however, whether sustained NRF2 in established HTS is protective, compensatory/pro-resolution, or persistence-permissive remains unresolved. Therapeutic testing should therefore discriminate among these possibilities rather than assume a late-stage requirement for NRF2 suppression.

This section therefore organizes candidate therapeutic strategies according to their molecular mechanism of action and the pathological process they engage, with representative interventions summarized in Table 2. Throughout, we apply the interpretive discipline established in Section 1: improved scar morphology, reduced collagen deposition, lower ROS, or decreased inflammation should not be taken as evidence of NRF2 action unless accompanied by nuclear NRF2 accumulation, coordinated ARE target-gene induction, or demonstrated NRF2 dependence [22], [23], [27], [28], [51]. This distinction is essential for separating true NRF2-directed effects from broader antioxidant, anti-inflammatory, antifibrotic, or ferroptosis-modifying actions.

### 5.1 Conditional stage- and mechanism-aware modulation

HTS-relevant pharmacology can be viewed as two mechanistically opposing perturbations within the proposed dual-degron framework rather than as fixed treatment strategies. NRF2 activators—typically electrophiles that modify reactive KEAP1 cysteines, including sulforaphane, fumarates such as dimethyl fumarate (DMF), and synthetic triterpenoids such as CDDO derivatives and RTA 408—have been reported to reduce scar- or fibrosis-associated readouts mainly in cutaneous fibrosis and wound-healing models that emphasize early, preventive, or pro-resolution contexts [24], [29], [36], [37], [140], [141], [144], [146], [147]. However, direct efficacy data in human HTS remain limited. By contrast, NRF2 suppression or destabilization reduces scar-associated endpoints, including scar elevation index, α-SMA, collagen I/III, and dermal thickness, in primary HTS-derived scar fibroblasts and rabbit-ear models that more closely approximate maturing or established scars [126], [127]. These findings are consistent with the hypothesis that sustained NRF2 activity may support persistence by reinforcing anti-ferroptotic defense in activated fibroblasts (Section 4.4). Accordingly, activation and suppression should be treated as experimental tests of competing hypotheses rather than as stage-prescribed therapeutic directions.

This division between activation and suppression is the operational consequence of the framework, but it should be interpreted as provisional rather than prescriptive. The activator and suppressor literatures differ in model system, scar stage, treatment timing, and endpoint selection, and they have not been compared head-to-head in stage-annotated human HTS. It therefore remains possible that NRF2 activation is beneficial even in some established-scar contexts, particularly if activity is compartment-specific, transient, or linked to resolution rather than persistence. Accordingly, NRF2 activation and suppression should be treated as competing experimental perturbations rather than stage-prescribed therapies, with their effects evaluated according to scar phenotype, cell-resolved NRF2 engagement, and disease-relevant scar endpoints.

### 5.2 Pharmacological modulation and the requirement to verify engagement

Evidence for direct NRF2 activators in HTS remains largely extrapolated from general NRF2 pharmacology and selected skin-context studies. Clinically used or clinical-stage NRF2 activators show that pharmacological pathway engagement is achievable in humans, but they should be interpreted as benchmark pharmacology rather than evidence of HTS efficacy [24], [37], [145], [148]. Sulforaphane, for example, has documented oral bioavailability and evidence of cutaneous NRF2 engagement, but controlled efficacy data in HTS are lacking [29], [140], [142], [143]. This distinction should be stated explicitly when such agents are used as mechanistic benchmarks.

Two mechanistic cautions constrain interpretation. First, many interventions attributed to NRF2 act through additional or alternative routes. A large literature reports improved scar or fibroblast outcomes after antioxidants, pleiotropic electrophiles, or repurposed drugs, and then attributes these effects to NRF2 on the basis of total NRF2 protein or HO-1 alone. This inference is weak because HO-1 can be induced through multiple NRF2-independent pathways, and total NRF2 abundance does not confirm nuclear localization, ARE binding, or transcriptional output [1], [38], [67]. Even dimethyl fumarate (DMF), a clinically relevant electrophilic NRF2 activator, has NRF2-independent anti-inflammatory actions, including protein succination and GAPDH inactivation; therefore, its effects should not be classified as NRF2-dependent unless supported by nuclear NRF2 readouts and coordinated ARE target induction [37]. Second, the pharmacological tools used to infer NRF2 dependence are themselves imperfect. Brusatol suppresses NRF2 nonspecifically through global translation inhibition, ML385 is more selective but still requires confirmatory testing, and bruceine D provides a scar-relevant indirect suppressor by degrading PIM1 and destabilizing NRF2 [51], [52], [126]. For this reason, pharmacological inhibition should ideally be paired with genetic *NFE2L2* perturbation before claims of NRF2 necessity are made.

Where ROS scavenging rather than NRF2 engagement is the intended intervention, N-acetylcysteine should not be used as the sole comparator. Cysteine donors with distinct pharmacokinetic and biochemical properties, such as N-acetylcysteine amide and γ-glutamylcysteine, can raise glutathione availability without necessarily engaging the KEAP1–NRF2 transcriptional axis. Such comparators help separate NRF2-dependent transcriptional effects from NRF2-independent redox buffering.

Ferroptosis-modulating agents require a higher evidentiary bar than general antioxidant or cytotoxicity claims. The four-criterion standard outlined in Section 4.4 should be applied: lipid-peroxidation accumulation, altered ferroptosis-defense systems such as GPX4 and SLC7A11/xCT, ideally ACSL4 or iron-related readouts, and mechanism-specific rescue with ferroptosis inhibitors or iron chelation. By this standard, bruceine D and tubeimoside I are the most relevant ferroptosis-focused scar-model interventions with NRF2-linked mechanisms [126], [127]. In contrast, dioscin, hypericin-mediated photodynamic therapy, and fraxinellone should be described as ferroptosis-associated or ferroptosis-targeting only to the extent that mechanism-specific rescue is demonstrated [129], [130], [131]. Cell-targeted erastin delivery using FAP-sEV microneedles provides an important proof-of-concept for selective ferroptosis sensitization in hypertrophic scar fibroblasts, but because it was not designed to test NRF2 engagement, it should be interpreted as support for local ferroptosis sensitization rather than as evidence of NRF2 dependence [128].

Natural products represent the largest and most heterogeneous candidate class. Many phytochemicals can activate or suppress NRF2 through electrophilic, metabolic, or stress-signaling mechanisms, while also modulating MAPK, NF-κB, TGF-β/Smad, mitochondrial function, autophagy, and ferroptosis independently of NRF2 [29], [89], [157]. Two requirements should therefore be applied. Mechanistically, attribution to NRF2 should require convergent evidence, including nuclear NRF2 localization or ARE-reporter activity, coordinated induction of at least two canonical NRF2 targets such as *NQO1* together with glutathione-axis genes, and ideally demonstrated NRF2 dependence by genetic or rigorously validated pharmacological perturbation. Chemically, the tested entity must be sufficiently defined: source, extraction method, active-component characterization, batch consistency, and dose normalization should be reported rather than treating an uncharacterized mixture as a mechanistically interpretable NRF2 modulator [22], [23], [28], [67].

### 5.3 Local delivery, safety, and conditional targeting

The conditional nature of NRF2 makes delivery strategy a mechanistic variable, not merely a pharmacokinetic one. Systemic NRF2 activation can engage phase I–III detoxification and efflux programs beyond the target tissue, alter drug handling, and modulate immune and stress-adaptation pathways. Experience from oncology further cautions that persistent NRF2 activation can confer cell-survival advantages and therapy tolerance [31], [158], [159], [160], [161], [162]. The clearest human safety signal comes from sustained high-level activation: the synthetic triterpenoid bardoxolone methyl (CDDO-Me), a potent NRF2 activator, led to early termination of the Phase III BEACON trial in patients with type 2 diabetes and stage-4 chronic kidney disease because of excess cardiovascular events, including heart failure [149]. This does not preclude NRF2-directed therapy, but it argues strongly against chronic systemic activation and supports local, stage-restricted delivery. Because HTS treatment courses can extend over weeks to months, even nominally local NRF2-directed therapy carries a theoretical local-to-systemic exposure risk, including the premalignant/malignant survival advantage noted above; this risk has not been quantified for HTS-directed NRF2 therapy and warrants exposure monitoring, local dosing limits, and long-term safety assessment before extended local use is recommended. Electrophilic activators such as sulforaphane, DMF, and CDDO derivatives warrant additional caution because their Michael-acceptor or alkylating chemistry can modify redox-sensitive proteins beyond KEAP1. Sulforaphane, for example, covalently modifies multiple cysteine-containing phosphatases, kinases, and metabolic enzymes, so its effects should not be assumed to be NRF2-restricted [37], [157]. This dose-dependent off-target reactivity further reinforces the rationale for confining electrophilic activity to the scar.

Because HTS is a localized lesion, local delivery offers a mechanistically attractive strategy: it can concentrate exposure within the scar while limiting systemic burden, building on familiar clinical modalities such as topical silicone, intralesional corticosteroids with or without 5-fluorouracil, laser-assisted approaches, and surgical revision [6], [7], [151], [163]. Topical delivery may be most appropriate for superficial lesions, but penetration into established scars is inconsistent because of altered barrier function, dense ECM, and variable vascularity. Intralesional injection bypasses some of these barriers but may distribute unevenly within stiff or heterogeneous scar tissue [6], [7], [163]. Microneedle and hydrogel platforms can improve dermal penetration and provide sustained local release [150], [152], [153], [154], [164], while the recent FAP-targeted sEV-erastin microneedle study illustrates how cell-directed delivery could be coupled to ferroptosis sensitization in scar fibroblasts [128]. These delivery platforms should be evaluated according to lesion characteristics, target compartment, and demonstrated pathway engagement rather than assigned to a fixed stage-specific NRF2 activation or suppression strategy.

These considerations point to a concrete and near-term-testable translational design rather than a generic call for “NRF2 modulation.” The most tractable first candidates are not novel botanicals but chemically defined, locally deliverable agents evaluated according to scar phenotype, target compartment, and demonstrated pathway engagement rather than chronological stage alone. An electrophilic activator with existing human cutaneous engagement data—sulforaphane or topical RTA 408 (Table 2), with their stated off-target caveats—could be used to test whether transient NRF2 activation dampens oxidative-inflammatory amplification in a defined scar context. Local, cell-directed ferroptosis sensitization using the FAP-targeted erastin-microneedle platform [128] could likewise test fibroblast vulnerability without assuming that a response establishes NRF2 dependence. Delivery can build directly on modalities already in clinical scar practice: fractional ablative laser-assisted delivery and microneedle or hydrogel platforms can improve penetration through barrier and dense-ECM constraints [6], [7], [150], [151], [152], [153], [154], [163], [164], but their use should be evaluated with appropriate platform controls rather than nominated as a fixed stage-specific combination regimen [151]. At every stage, the go/no-go criterion should be pathway engagement read by the E2P panel on paired pre- and post-treatment microsamples, not reduction in oxidative markers alone.

Deliberate induction of ferroptosis in scar fibroblasts *in vivo* carries risks that the preclinical literature has not yet addressed and that any translational program must confront directly. Ferroptosis is intensely pro-inflammatory through release of damage-associated molecular patterns, and could therefore paradoxically reignite the fibroinflammatory loop it is intended to resolve; bystander ferroptosis in keratinocytes, endothelium, or adnexal structures could impair re-epithelialization and precipitate wound dehiscence or ulceration; and the lipophilic, electrophilic chemistry of several candidate inducers, including dioscin, bruceine D, and tubeimoside I, lacks route-specific tolerability, barrier-recovery, and off-target-cytotoxicity data [126], [127], [129]. Cell-directed delivery, such as FAP-targeting of the affected fibroblast population [128], is the principal mitigation and should be evaluated against these specific endpoints—re-epithelialization rate, barrier-function recovery, local inflammatory markers, and wound integrity—rather than against scar-reduction efficacy alone. The transient, reversible barrier effects reported for topical simvastatin in the rabbit-ear model [165] illustrate both the relevance of this concern and a feasible assay for it.

Two further mechanistic cautions are essential for interpreting local-delivery studies. First, platform and payload effects must be separated, because microneedles, hydrogels, extracellular vesicles, and laser-assisted delivery can themselves alter inflammation, matrix remodeling, wound tension, and redox tone. Second, any NRF2-directed payload should be evaluated using the revised E2P domains: disease proximity/model relevance, NRF2 pathway engagement including nuclear NRF2 localization and a coherent multi-gene regulon, causal NRF2 dependence where feasible, linkage to disease-relevant scar phenotypes, and independent replication/methodological quality. Reduced ROS, improved scar texture, or lower collagen content alone does not establish NRF2-dependent target engagement.

The most defensible therapeutic framework is therefore a conditional, locally controlled NRF2 research strategy rather than a fixed stage-prescribed activation-versus-suppression regimen (Fig. 3). Preventive or early studies may test whether dampening excessive oxidative-inflammatory amplification without suppressing physiological repair signals is beneficial [84], [166]. Established-scar studies may test whether reducing fibroblast stress tolerance, increasing ferroptosis sensitivity, remodeling ECM architecture, and weakening mechanical reinforcement improves outcome, potentially complementing approaches that exploit myofibroblast fate plasticity, including deactivation, apoptosis, senescence, and reprogramming [167]; these remain experimental hypotheses rather than treatment assignments. Realizing such a conditional strategy in patients requires prospective stratification by objective scar phenotype, target cellular compartment, and local pathway engagement rather than chronological time alone, since HTS is heterogeneous across cause (burn, surgical, traumatic), anatomic tension environment, skin phototype, and time since injury; this is precisely the prospective stratification that the stage- and compartment-resolved map proposed in Sections 3.2, 6, together with a minimally invasive surrogate readout, would enable. The current literature often lacks route-specific tolerability data, systemic-exposure characterization, local-irritation and barrier monitoring, off-target cytotoxicity assessment, and recovery data, particularly for potent bioactives and ferroptosis modulators such as bruceine D, tubeimoside I, and dioscin [126], [127], [129]. Topical simvastatin in a rabbit-ear HTS model is instructive in this regard, because it reported transient barrier effects that reversed after withdrawal [165]. Across approaches, therapeutic claims should be tied to verified pathway engagement, scar-stage annotation, local exposure, tolerability, and standardized HTS endpoints. These requirements distinguish true NRF2-directed therapy from nonspecific antioxidant, anti-inflammatory, antifibrotic, or cytotoxic intervention [6], [27], [150], [164]. Representative interventions, their proposed mechanisms, and their evidence status are summarized in Table 2.

## 6 Outlook: the mechanistic experiments that would resolve directionality

The central question of this review is whether NRF2 function differs across HTS stage and cellular context, and whether any late-scar persistence-permissive activity is causal rather than compensatory. The dual-degron organization is a working model assembled from established Neh2–KEAP1 and Neh6–GSK-3β/β-TrCP mechanisms [21], [22], [23], [24], [61], [62], [63], [64], not a directly observed temporal switch. A focused set of studies, summarized in Fig. 2, can distinguish NRF2 deficiency, compensatory activation, and persistence-permissive activity without presupposing which model is correct.

**Fig. 2:**
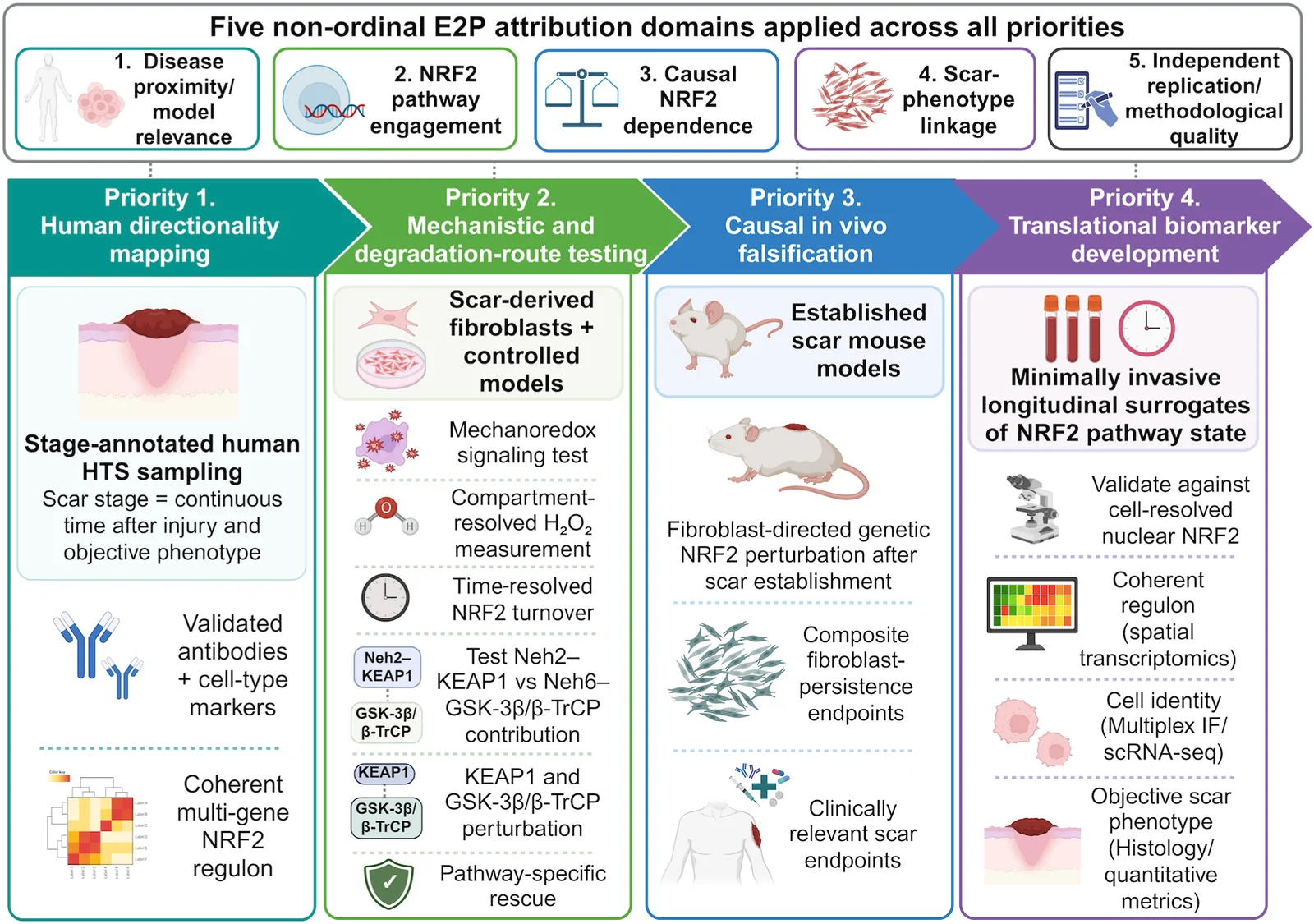
**An engagement-to-phenotype (E2P) research roadmap for resolving NRF2 directionality in hypertrophic scarring.** A research evidence-generation pipeline—not an individual-patient treatment algorithm—organized around five non-ordinal E2P attribution domains that are applied across the roadmap: disease proximity/model relevance, NRF2 pathway engagement, causal NRF2 dependence, scar-phenotype linkage, and independent replication/methodological quality. Priority 1, human directionality mapping: quantify nuclear NRF2 with validated antibodies, cell-type markers, and a coherent multi-gene NRF2 regulon in stage-annotated human HTS, with scar stage defined by continuous time together with objective phenotype. Priority 2, mechanistic and degradation-route testing: use scar-derived fibroblasts and controlled models to test mechanoredox signaling and the proposed Neh2–KEAP1 versus Neh6–GSK-3β/β-TrCP contribution using defined stiffness, compartment-resolved H_2_O_2_ measurements, time-resolved NRF2 turnover, KEAP1 and GSK-3β/β-TrCP perturbation, and pathway-specific rescue. Priority 3, causal *in vivo* falsification: test fibroblast-directed genetic NRF2 perturbation after scar establishment using composite fibroblast-persistence and clinically relevant scar endpoints. Priority 4, translational biomarker development: develop minimally invasive longitudinal surrogates of NRF2 pathway state and validate them against cell-resolved nuclear NRF2, a coherent regulon, cell identity, and objective scar phenotype before using them to support local intervention studies. Together, these steps are designed to distinguish NRF2 deficiency, compensatory/pro-resolution activation, and persistence-permissive NRF2 activity without presupposing which model is correct.

**Fig. 3:**
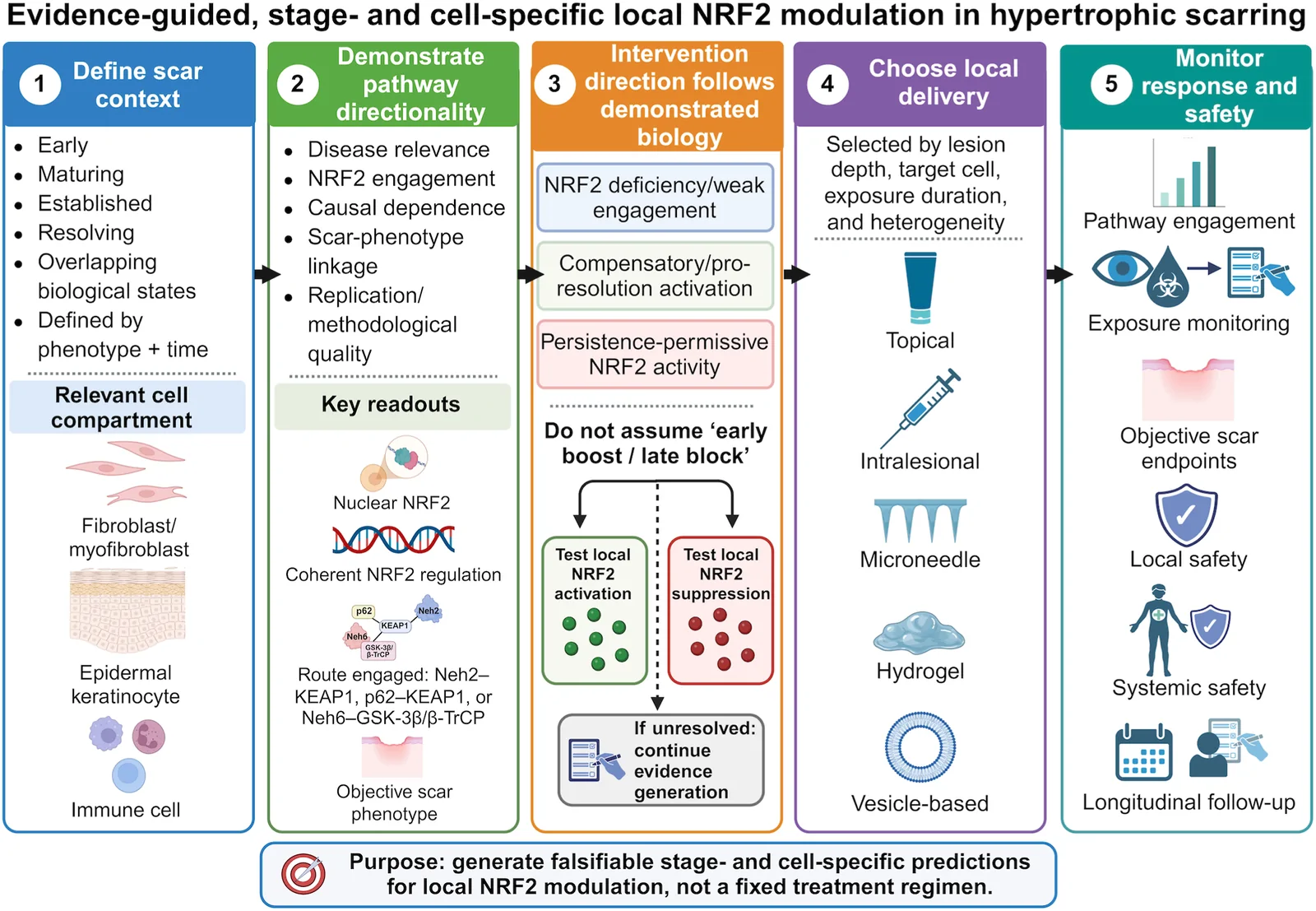
**Stage-aware, locally controlled NRF2 modulation in hypertrophic scarring.** A conditional research framework for locally controlled NRF2 modulation in HTS, linking the mechanistic model in Fig. 1 and the evidence-generation roadmap in Fig. 2 through the five non-ordinal E2P attribution domains. Early, maturing, established, and resolving scars are overlapping biological states defined by phenotype as well as time. The cell compartments shown are representative; the full set recommended for stratified analysis (fibroblast/myofibroblast, keratinocyte, macrophage, and endothelial) is specified in Section 3.1. Local NRF2 activation or suppression should be investigated only after the direction of pathway activity is demonstrated in the relevant cell compartment and linked to scar phenotype. Delivery options include topical, intralesional, microneedle, hydrogel, and vesicle-based approaches, selected according to lesion depth, target cell, exposure duration, and heterogeneity. Because human activator-versus-suppressor directionality remains unresolved, the framework is intended to generate falsifiable stage- and cell-specific predictions rather than prescribe an ‘early boost/late block’ regimen. Long-duration local treatment also requires exposure monitoring and local/systemic safety assessment.

NRF2 output is strongly tissue-, cell-state-, and upstream-pathway-dependent [24], [39], [40], and evidence from other organ systems—cited here only as non-HTS context, not as direct HTS evidence—illustrates this range. Adipose-tissue-specific *Nfe2l2* loss-of-function in obese mice has been reported to improve an inflammatory-metabolic phenotype partly through suppression of cGAS-STING signaling [168], providing an example in which *Nfe2l2* loss, rather than gain, is protective in that tissue. In ovarian cancer, a CEBPB-NRF2-DUSP1/MAPK axis has been linked to enhanced proliferation and antioxidant capacity [158], [162], [169], whereas in osteoarthritic chondrocytes a Sirt6-Keap1/Nrf2/HO-1 axis has been linked to DNA-damage repair and protection from cellular senescence [170], illustrating divergent, cell-state-dependent consequences of the same core pathway. In heart failure, recent syntheses generally emphasize impaired Nrf2 signaling and the potential cardioprotective consequences of restoring pathway activity [171], further illustrating the organ-specific context of NRF2 function. In cardiac hypertrophy, a ZDHHC18–YTHDF2–USP13 pathway has been reported to converge through USP13–SNX13 on Nrf2/ferroptosis signaling [172], providing another example of upstream context-specific regulation of NRF2 outside HTS. None of these findings are treated as HTS-specific; they are included to show that the direction of NRF2's effect in established HTS cannot be assumed from its role in other tissues and must be tested directly.

The first and most decisive experiment is a stage- and compartment-resolved map of NRF2 activity in human HTS tissue. Imaging mass cytometry or multiplexed antibody imaging can quantify validated nuclear NRF2 together with fibroblast, myofibroblast (α-SMA), macrophage, endothelial, and keratinocyte markers at single-cell resolution *in situ*, while spatial transcriptomics can assess a coordinated NRF2 regulon, including *NQO1*, *GCLC*, *GCLM*, *GSR*, and *SLC7A11*/xCT, rather than relying on *NFE2L2* mRNA or HO-1 alone [24], [40], [43], [73]. Imaging mass spectrometry has also been used to characterize molecular changes during burn wound healing [173], but this evidence does not by itself establish single-cell fibroblast states or NRF2 pathway activity. Single-cell transcriptomic studies have identified fibroblast heterogeneity in human HTS [80] and keloid tissue [81]. Keloid-derived fibroblast states should not be interpreted as HTS-specific unless independently validated in HTS samples. Accordingly, whether particular NRF2-high, stress-adapted, or profibrotic fibroblast states are selectively enriched in HTS remains unresolved. AI-assisted digital pathology may support objective segmentation and pattern integration across nuclear NRF2, collagen architecture, vascularity, scar elevation, and biomechanical features, but it cannot substitute for pathway-specific validation. To make the temporal axis interpretable, scar stage should be treated as overlapping/continuous and defined by time since injury together with objective scar phenotype rather than by rigid temporal cutoffs. Resolving HTS and mature normotrophic scars should be included as comparators where feasible, and etiology, anatomical tension, age, phototype, and treatment history should be pre-specified. Nuclear NRF2 antibody validation using appropriate positive/negative and orthogonal controls should also be required. Applied this way, the analysis would determine whether nuclear NRF2 rises, falls, or redistributes in myofibroblasts as scars mature, against the discriminating signatures set out in Section 3.2. This is the single dataset on which the NRF2–HTS directionality question most directly turns, and it directly addresses the central gap identified in Section 3.2 [19], [40], [43], [73].

The second experiment is a direct test of the mechanoredox prediction developed in Section 4.5. Using stiffness-tunable substrates, studies should determine whether increasing matrix stiffness raises nuclear NRF2 in scar fibroblasts through FAK–PI3K–AKT–GSK-3β signaling, even without a proportional increase in oxidative stress, and whether matrix softening or FAK/AKT inhibition reverses this effect. A positive result would establish NRF2 as a mechano-transcriptional output of the stiff scar niche [57], [71], [133], [134]. Because this prediction depends on distinguishing mechanical stabilization from redox-driven activation, the design must meet the discriminating criteria specified in Section 4.5: stiffness-dependent nuclear NRF2 must persist under an antioxidant clamp and be abolished by AKT or GSK-3β manipulation but not by KEAP1 knockdown. The redox readout must accordingly be compartment-resolved. Genetically encoded H_2_O_2_ biosensors such as roGFP2-Orp1 or HyPer7, together with explicit GSH/GSSG measurement and NADPH/NADP^+^ sensors such as iNap or SoNar, would be far more informative than bulk oxidative-marker staining—and the NADPH/NADP^+^ readout in particular is what would test the NADPH-reductive-shift prediction of Section 2.3, distinguishing a reductively shifted, persistence-permissive myofibroblast from a still-oxidatively-stressed compensatory one.

A complementary time-resolved degron experiment is required before a temporal switch can be claimed. Scar-derived fibroblasts should be followed under defined oxidative/electrophilic stimulation and graded stiffness/growth-factor conditions with NRF2 half-life or pulse-chase measurements, nuclear NRF2 and multi-gene regulon readouts, KEAP1 perturbation, GSK-3β/β-TrCP manipulation, and Neh6-sensitive reporter or mutant constructs where feasible. β-TrCP–NRF2 interaction/degradation assays and pathway-specific rescue should distinguish changes in Neh6 turnover from changes in Neh2–KEAP1 control. Equivalent measurements in the same cell population over time are required to demonstrate a true transition rather than infer one from separate experiments.

The third experiment is an *in vivo* falsification test. Fibroblast-directed genetic NRF2 ablation should be performed in an established scar model, such as the rabbit-ear HTS model, after scar formation rather than only during early injury. If persistence-permissive NRF2 activity is operative, fibroblast-restricted NRF2 loss should reduce a composite persistence phenotype including α-SMA together with matrix/activation markers such as COL1A1/collagen I and POSTN (with COMP/COL11A1 where appropriate), plus collagen organization, contractility, scar elevation index, and mechanical stiffness. Failure to improve these established-scar endpoints would argue against persistence-permissive NRF2 as a clinically relevant driver in mature HTS. As set out in Section 4.4, this experiment is the pre-specified test that would move the persistence-permissive model from hypothesis to supported claim.

A fourth priority is translational rather than purely mechanistic: developing a minimally invasive, longitudinal readout of NRF2 pathway state, because serial full-thickness scar biopsy is impractical and ethically constrained at the scale a staged clinical program would require. Candidate surrogates include scar interstitial-fluid sampling (for example by microdialysis) for lipid-peroxidation and iron markers, tape-stripping for epidermal ARE-target transcripts, and non-invasive optical or elastographic imaging correlated against the spatial map generated in the first experiment. These approaches should be regarded as candidate surrogates rather than validated NRF2 biomarkers for HTS and would require paired validation against cell-resolved nuclear NRF2 and objective scar phenotype before replacing tissue sampling. Without such a surrogate, the paired pre- and post-treatment sampling on which the engagement-confirmation steps of Fig. 2 depend remains a practical bottleneck.

Three further mechanistic questions follow directly from this roadmap. First, do the glutathione-independent FSP1–CoQ10 (and DHODH) arm and the ACSL4, rather than ACSL1, ferroptosis-sensitivity axis contribute to ferroptosis resistance in activated scar fibroblasts, given that resistance running through the glutathione-independent arm would render NRF2 suppression alone insufficient (Section 4.4) [69], [115], [116], [120], [121], [122]. Second, do post-translational NRF2 inputs beyond KEAP1 and Neh6, including p62-mediated KEAP1 sequestration and OGT-mediated O-GlcNAcylation, operate in defined HTS stages and cellular compartments [66], [67], [68], [89]. Third, does NRF2 activation segregate with specific pathological fibroblast subclusters resolved by single-cell transcriptomics, and does any NRF2-high subset map to myofibroblast persistence rather than remodeling competence [80], [81], [82], [83].

These questions are experimentally tractable. Scar-derived fibroblasts can be cultured on stiffness-tunable hydrogels, exposed to defined TGF-β/NOX4/AKT or ferroptosis stimuli, and perturbed genetically at NFE2L2, KEAP1, GSK-3β, p62, ACSL4, SLC7A11, GPX4, FSP1, or OGT. Endpoints should include nuclear NRF2, coordinated ARE target induction, lipid-peroxidation and ferroptosis-rescue assays, a composite fibroblast-persistence phenotype combining α-SMA with COL1A1/collagen I and POSTN (with COMP/COL11A1 where appropriate), CTGF, contractility, collagen organization, apoptosis, senescence, and scar-fibroblast survival. In tissue and animal models, these molecular endpoints should be linked to clinically meaningful scar outcomes, including scar elevation, collagen organization, pliability, vascularity, pigmentation, and validated clinical instruments such as the Patient and Observer Scar Assessment Scale and the Vancouver Scar Scale, together with objective biomechanical or imaging measures [46], [47], [174], [175], [176].

## 7 Conclusions

HTS arises from sustained coupling among redox imbalance, inflammation, fibroblast activation, mechanical reinforcement, and pathological ECM remodeling. The evidence reviewed here supports positioning NRF2 as a major redox-regulatory node in this process, but not as a simple antifibrotic switch. Its role is conditional, depending on scar stage, cell type, mechanical niche, degradation route, and duration of pathway engagement. The most important unresolved issue is therefore not whether NRF2 participates in scar biology, but whether established HTS reflects insufficient NRF2 activity, compensatory NRF2 activation that remains net pro-resolution, or persistence-permissive NRF2 activity that increases stress tolerance of already activated myofibroblasts. We advance the persistence-permissive interpretation as an explicit, testable hypothesis rather than as an established conclusion; however, support for this interpretation remains narrow and partly single-group and should not supersede the broader protective genetic evidence from wound and organ-fibrosis models. This evidential asymmetry and the pre-specified falsification test are detailed in Section 4.4.

The contribution of this review is a mechanistic, stage- and compartment-resolved account of where NRF2 sits within the redox pathobiology of HTS. In early repair, transient redox-driven NRF2 activation through the Neh2–KEAP1 arm may protect tissue by limiting oxidative-inflammatory amplification and excessive injury. In established scars, however, sustained NRF2 activity driven by growth-factor and mechanical signaling or, separately, by autophagy-linked p62–KEAP1 signaling may support myofibroblast metabolic fitness, stress tolerance, and ferroptosis resistance—while, as the reductive-stress argument in Section 2.3 notes, potentially exposing an intrinsic vulnerability of the chronically NRF2-high myofibroblast. The proposed dual-degron model considers whether the redox-sensitive Neh2–KEAP1 arm contributes more during early injury whereas the KEAP1-independent Neh6–GSK-3β/β-TrCP arm may contribute more in established scars; however, this temporal shift has not been directly demonstrated in HTS. This framework generates concrete predictions for mechanoredox signaling, ferroptosis biology, and stage-aware therapeutic modulation.

Reduced ROS, lower collagen, improved scar morphology, or decreased inflammation do not by themselves prove NRF2 involvement, because antioxidant agents, cytoprotective compounds, ferroptosis modulators, and delivery platforms can produce similar outcomes through NRF2-independent mechanisms. Claims of NRF2 involvement should therefore require nuclear NRF2 localization, coordinated ARE target induction, demonstrated NRF2 dependence where feasible, and linkage to disease-relevant scar endpoints.

Translation should prioritize stage-aware, locally controlled modulation conditioned on demonstrated pathway directionality, cell type, scar phenotype, and local target engagement rather than chronic, generic NRF2 activation or a fixed early-versus-late treatment strategy. Ferroptosis-targeting claims should be reserved for studies showing lipid-peroxidation accumulation together with mechanism-specific rescue. The most immediately achievable advance is to map nuclear NRF2 with cell-type co-markers, using the spatial and single-cell methods now available, in well-annotated human HTS biopsies across scar maturation defined by time together with objective phenotype. This dataset is feasible within existing burn and plastic-surgery research infrastructures and would directly adjudicate the directional question central to NRF2's role in HTS.

## CRediT authorship contribution statement

**Weerawon Thangboonjit:** Writing – review & editing, Writing – original draft, Investigation, Conceptualization. **Mutita Pluempreecha:** Writing – review & editing, Visualization, Project administration, Investigation. **Tanyapohn Soingam:** Writing – review & editing, Visualization. **Uraiwan Panich:** Writing – review & editing, Writing – original draft, Supervision, Resources, Funding acquisition, Conceptualization.

## Declaration of generative AI and AI-assisted technologies in the manuscript preparation process

During the preparation of this work, the authors used ChatGPT (OpenAI) to assist with language editing, grammar refinement, and improvement of manuscript clarity. After using this tool, the authors reviewed and edited the content as needed and take full responsibility for the content of the published article.

## Funding

This work was supported by Mahidol University [Fundamental Fund: fiscal year 2026 by National Science Research and Innovation Fund (NSRF)].

## Declaration of competing interest

The authors declare that they have no known competing financial interests or personal relationships that could have appeared to influence the work reported in this paper.

## Data Availability

No primary datasets were generated or analyzed in this review. All data discussed are available in the cited published articles.
