# Supplementary material for: NRF2 in hypertrophic scarring: A stage-dependent redox hub poised between resolution and persistence
Source: Redox Biol. 2026 Oct 27;98:104460. doi: 10.1016/j.redox.2026.104460 (PMC13744350; doi:10.1016/j.redox.2026.104460)
Supplement: Supplementary file 1 [file mmc1.docx]

**Supplementary File S1**

**Literature-search documentation**

**Journal:** Redox Biology

**Manuscript title:** NRF2 in Hypertrophic Scarring: A Stage-Dependent Redox Hub Poised Between Resolution and Persistence

**Authors:** Thangboonjit W., Pluempreecha M., Soingam T., Panich U.

**Supplementary File S1.** Narrative literature-search documentation for the Redox Biology review, including the databases searched, the search window, the core search terms, the targeted search supporting the Section 3.2 negative finding, the provenance of the Table 1 and Table 2 disease-source labels, a rapid pre-submission update check, the reference-list consolidation performed at final revision, and the limitations of the non-systematic search approach.

**Purpose.** This review is a non-systematic narrative review. The documentation below is provided to support reproducibility and, in particular, to allow verification of the negative finding reported in Section 3.2 of the manuscript: we identified no indexed study providing quantitative nuclear NRF2 immunofluorescence with cell-type co-localization in stage-annotated human hypertrophic scar (HTS) tissue. This absence reflects a limit of the indexed, peer-reviewed literature search rather than proof that no such data exist; non-English-language reports, preprints, theses, and unannotated pathology-archive material were not reliably captured (see Section 5). The search was not conducted as a staged systematic review; no PRISMA flow diagram is provided, and no prospectively logged screening counts are claimed. The documentation is therefore presented as a transparent narrative-search record rather than a systematic-review report.

**1. Databases, dates, and approach**

**Databases searched:** PubMed/MEDLINE and Scopus were searched in parallel, with records combined iteratively. A rapid pre-submission update check was also performed using web-accessible searches for newly indexed scar/NRF2/ferroptosis records. This check was not intended as a formal systematic search and was not included in prospectively logged screening counts.

Search window: The core narrative search covered publications from 1 January 2000 to 31 May 2026. A rapid pre-submission update search was conducted on 20 June 2026. A further targeted web-accessible update check was conducted on 19 October 2026; PubMed/MEDLINE and Scopus were not rerun as full database searches for this update. Accordingly, the negative finding is current only within the limits of these searches and should be reassessed as new literature becomes available.

Currency limitation for this resubmission: the core targeted database search and negative finding were last based on the PubMed/MEDLINE and Scopus search completed through 31 May 2026, with a rapid update on 20 June 2026. The 19 October 2026 check was a targeted web-accessible update, not a full rerun of both databases. It identified an independent gain-/loss-of-function study in human HTS fibroblasts, now cited as Reference [35]; this study does not meet the strict criterion for stage-annotated human HTS tissue with quantitative nuclear NRF2 immunofluorescence and cell-type co-localization. The reported qualifying-record count therefore remains 0 under that criterion, but should be reassessed after a full database update if required.

Approach: Iterative topic searches combined the core concept terms below (Section 2), supplemented by manual screening of reference lists from recent reviews on NRF2, ferroptosis, scar biology, and mechanotransduction. Studies were prioritized when they provided cell-resolved NRF2 readouts in scar-relevant tissue or fibroblasts; reviews and mechanistic studies in non-scar systems were included only to support biological plausibility. Records were de-duplicated informally across databases during the iterative search process rather than as a formal staged step. The rapid 20 June 2026 update identified one additional relevant 2026 scar-ferroptosis record that was added to the manuscript for currency; it did not meet the stage-annotated nuclear NRF2 immunofluorescence criterion. The 19 October 2026 targeted web-accessible update identified an independent Nrf2 gain-/loss-of-function study in human HTS fibroblasts [35]. It provides in-vitro functional evidence but does not report stage-annotated human scar tissue with quantitative nuclear NRF2 immunofluorescence and cell-type co-localization, so it does not change the Section 3.2 negative finding.

**Disease-source provenance (Tables 1 and 2).** The disease-source labels applied throughout Tables 1 and 2 of the manuscript were assigned by reading the original Methods of each cited study, using the definitions stated in the Table 1 footnote: “Human HTS”, direct human hypertrophic-scar tissue; “primary HTS fibroblast”, cells derived from hypertrophic scar; “HTS-like”, animal models reproducing hypertrophic-scar features; “keloid/mixed scar”, material that is not treated as HTS-specific; and “non-HTS mechanistic”, evidence used to support biological plausibility rather than disease-specific proof. These labels are a transparency device intended to prevent over-generalization of non-HTS mechanistic evidence to human HTS and do not constitute a graded quality score.

**2. Core concept terms (combined iteratively with Boolean AND/OR)**

**Disease/tissue:** “hypertrophic scar”, “hypertrophic scarring”, “hypertrophic scars”, “keloid”, “cutaneous fibrosis”

**Pathway:** “NRF2”, “NFE2L2”, “KEAP1”, “antioxidant response element”, “ARE”

**Redox/cell death:** “oxidative stress”, “ferroptosis”, “lipid peroxidation”

**Crosstalk:** “p62”, “SQSTM1”, “TGF-β”, “Smad”, “NF-κB”, “mechanotransduction”

**Delivery:** “microneedle”, “hydrogel delivery”

**3. Targeted search underlying the Section 3.2 negative finding**

The targeted search used to test for the absence of cell-resolved nuclear NRF2 immunofluorescence in stage-annotated human HTS tissue was:

*((“hypertrophic scar” OR “hypertrophic scarring” OR “hypertrophic scars”) AND (“NRF2” OR “NFE2L2” OR “KEAP1” OR “antioxidant response element” OR “ARE” OR “oxidative stress” OR “4-HNE” OR “8-OHdG” OR “malondialdehyde” OR “MDA”))*

**Screening criterion.** Retrieved records were screened for the simultaneous presence of three features in human HTS tissue: quantitative nuclear NRF2 immunofluorescence, cell-type co-localization, and scar-stage annotation.

**Outcome.** No retrieved indexed record reported quantitative nuclear NRF2 immunofluorescence with cell-type co-localization in stage-annotated human HTS tissue. Records reporting total NRF2 protein abundance, single antioxidant markers (e.g., HO-1, NQO1), general oxidative-stress readouts (4-HNE, 8-OHdG, MDA), ferroptosis-associated endpoints, or in vitro immunofluorescence without scar-stage annotation were present but did not satisfy the criterion. As noted in Section 5, this outcome is bounded by the indexed-database search strategy and should not be read as establishing that no such data exist anywhere in the literature.

Search-currency note. The core search closed on 31 May 2026, the rapid update check was completed on 20 June 2026, and a targeted web-accessible update check was conducted on 19 October 2026. The October check was not a full rerun of PubMed/MEDLINE and Scopus. Any future verification should include a full database update and screen literature published or indexed after the last full database search.

**4. Retrieval and screening counts**

Because the literature was searched iteratively as a critical narrative review rather than as a staged systematic screen, per-step record counts were not prospectively logged. This is an inherent limitation of the narrative-search approach. The two figures that can be stated for the current manuscript are the number of cited records (176) and the number of records meeting the criterion for stage-annotated nuclear NRF2 immunofluorescence in human HTS tissue (0); these are summarized in the table below.

The current reference list contains 176 cited records, identified through PubMed/MEDLINE, Scopus, manual reference-list screening, and update checks. This total reflects several consolidation and revision steps. First, an earlier draft list was reduced to 154 by merging duplicate entries that had appeared under more than one reference number and removing a small number of records no longer cited in the text. Three NADPH oxidase 4 (NOX4) references were then added to provide correct primary-literature support for the TGF-β1/Smad3–NOX4 claims, raising the total to 157. Four further references were then added during table revision—a primary NRF2 O-GlcNAcylation study and clinical references for dimethyl fumarate, intralesional 5-fluorouracil, and topical RTA 408—raising the total to 161. Finally, four references were added during revision of Section 4 to deepen the redox-chemistry and directionality discussion, raising the total to 165. A further ten references were added during the final reviewer-driven revisions to support myofibroblast fate plasticity, cross-system NRF2 context, spatial/imaging approaches, and scar-outcome assessment, bringing the total to 175. One additional independent study of Nrf2 gain-/loss-of-function in human HTS fibroblasts was subsequently added as Reference [35], bringing the current reference count to 176. This study does not meet the strict stage-annotated nuclear NRF2 immunofluorescence criterion, so the qualifying-record count remains 0.

| **Search/screening stage** | **Count** | **Notes** |
| --- | --- | --- |
| Records identified (combined PubMed/MEDLINE + Scopus queries) | Not logged | Iterative narrative search; per-step counts not prospectively recorded |
| After de-duplication | Not logged | De-duplication performed informally across iterative search rounds |
| Title/abstract screened | Not logged | Not prospectively logged |
| Full text assessed for eligibility | Not logged | Not prospectively logged |
| Included and cited in manuscript | 176 | Current revised reference list; an earlier draft was reduced to 154 by merging duplicate entries and removing records no longer cited, then increased by three NOX4 references, four references added during table revision, four added during Section 4 revision, ten added during final reviewer-driven revisions, and one independent human HTS fibroblast gain-/loss-of-function study [35]. |
| Rapid update checks (20 June and 19 October 2026) | 2 added cited records; 0 qualifying records | One relevant record was added at each update. The October check was web-accessible and targeted, not a full PubMed/MEDLINE and Scopus rerun; neither record met the tissue-level nuclear NRF2 IF + cell-type co-localization + stage-annotation criterion. |
| Qualifying records: nuclear NRF2 IF + cell-type co-localization + stage-annotated human HTS tissue | 0 | Basis of the Section 3.2 negative finding under the stated criterion; full PubMed/MEDLINE and Scopus search through 31 May 2026, with update checks on 20 June and 19 October 2026. The October check was not a full database rerun. |

**5. Limitations of this documentation**

The absence of per-step record counts is a recognized limitation of iterative narrative searches. The two current findings reported above—176 cited records and no retrieved study meeting all three criteria in human HTS tissue (quantitative nuclear NRF2 immunofluorescence, co-localization with a specified cell type, and scar-stage annotation)—can be checked against the current manuscript and the stated search approach. The negative finding was last based on the full PubMed/MEDLINE and Scopus search through 31 May 2026 and the rapid update on 20 June 2026; the targeted web-accessible check on 19 October 2026 identified a human HTS fibroblast gain-/loss-of-function study [35] but no record meeting all three tissue-level criteria. The October check was not a full database rerun, so the absence finding should not be described as a comprehensive search current through 19 October 2026. A full database update is recommended if this question is revisited.

A further limitation concerns search coverage rather than search process: PubMed/MEDLINE and Scopus do not reliably index non-English-language journals, regional venues, preprints, theses, or pathology-archive material, and a meaningful share of the ferroptosis-in-scar literature originates from groups publishing partly in such venues. The negative finding in Section 3.2 of the manuscript should therefore be read as an absence within the indexed, peer-reviewed literature rather than a definitive absence in the literature at large.
